# Calix[6]arene-based atropoisomeric pseudo[2]rotaxanes

**DOI:** 10.3762/bjoc.14.186

**Published:** 2018-08-14

**Authors:** Carmine Gaeta, Carmen Talotta, Placido Neri

**Affiliations:** 1 Dipartimento di Chimica e Biologia " A. Zambelli", Università di Salerno, Via Giovanni Paolo II 132, 84084 Fisciano (Salerno), Italy

**Keywords:** atropoisomers, calixarene, conformation, pseudorotaxane, social isomerism

## Abstract

Some examples of atropoisomeric pseudorotaxanes in which the isomerism arises by the different conformations adopted by the wheel are reported here. Upon threading hexahexyloxycalix[6]arene **1** with ammonium axles **2^+^** or **3^+^**, bearing biphenyl or trifluoromethylbenzyl moieties, respectively, two atropoisomeric pseudorotaxanes were formed in which the calix[6]-wheel **1** adopts the *1,2,3-alternate* and *cone* conformations. The interconversion between them cannot be obtained by simple rotation around the ArCH_2_Ar bonds of the calixarene wheel, which is blocked by the presence of the axle inside its cavity. Therefore, it can only be obtained through a mechanism of de-threading/re-threading of the axle. In all the examined cases, the *1,2,3-alternate* and *cone* atropoisomers are, respectively, the kinetic and the thermodynamic ones.

## Introduction

Mechanomolecules [1–4], such as rotaxanes and catenanes show interesting properties as nanodevices for catalysis [5–8], recognition, and sensing [9–13]. Beyond these ascertained potentialities, interpenetrated architectures show fascinating structures that still stimulate the imagination of scientists.

An amazing aspect of rotaxanes and catenanes is their ability to adopt novel forms of isomerism. More in detail, (pseudo)rotaxane or catenane architectures can show novel stereoisomeric forms as a result of the "social" [14] relationship between their components.

**Figure 1:**
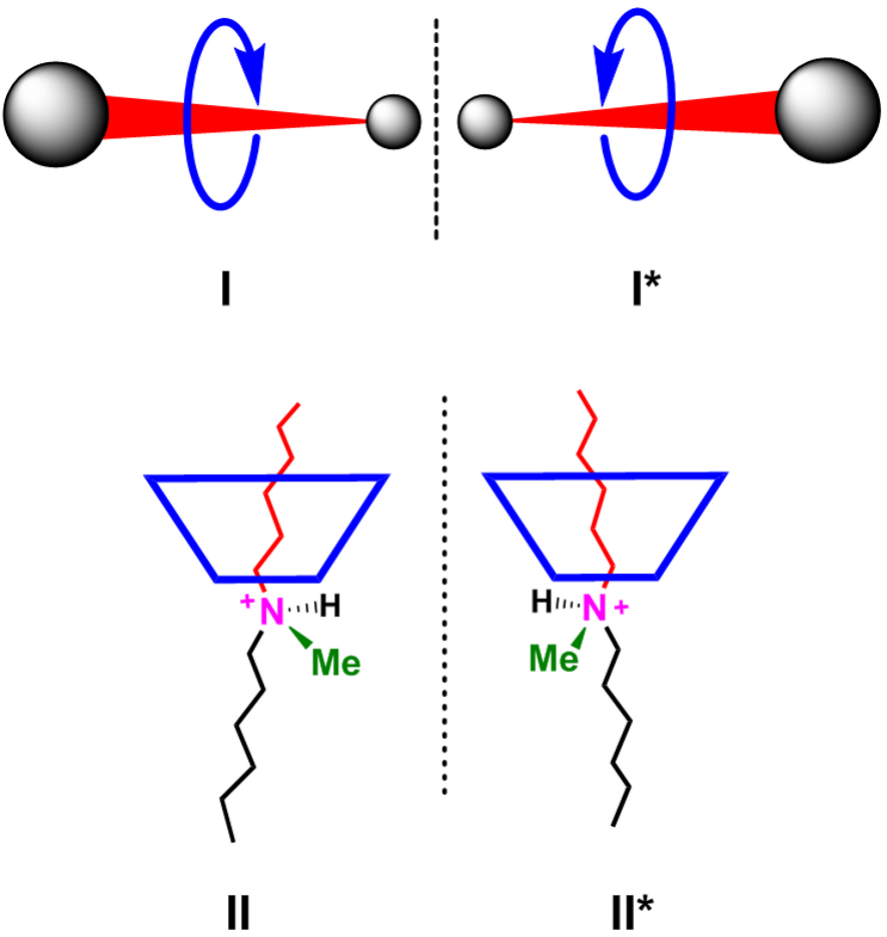
Cartoon representation of the chiral rotaxane of the Goldup group [15–16] (**I** and I*) and of the chiral pseudorotaxane (**II** and II*) reported by our group [17].

Recently, Goldup’s group assembled a mechanically planar chiral rotaxane [15–16] (**I** and I*, Figure 1) consisting of achiral components. The combination of a macrocycle with rotational asymmetry and a directional thread with non-equivalent ends is the cause of chirality in this example (Figure 1). Interestingly, our group showed that a chiral pseudorotaxane can be generated upon threading a tertiary ammonium axles in a directional (non-flat) calixarene-wheel (**II** and II*, Figure 1) [17]. In this case the chirality is created by the directionality of the calixarene wheel in a *cone* conformation, which differentiates the two alkyl chains around the prochiral ammonium center.

**Figure 2:**
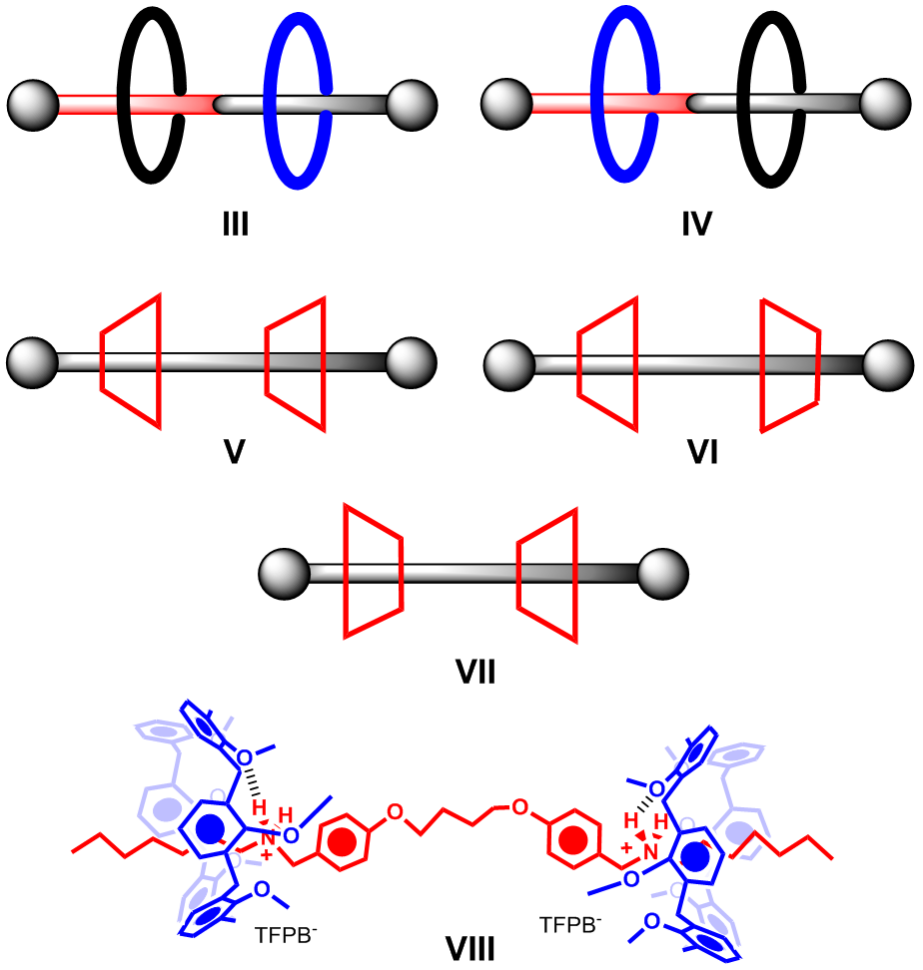
Cartoon representation of the rotaxane sequence isomers reported by Leigh [17] (**III** and **IV**) and of the pseudorotaxane sequential stereoisomers (**V**–**VII**) reported by our group [19–21].

In 2010, for the first time, an example of sequence isomerism was reported by Leigh’s group [18], caused by two different flat wheels that can be located differently along a directional thread **III** and **IV** (Figure 2). As an evolution of this concept, we envisaged a sequence stereoisomerism if two directional non-flat wheels, such as calixarenes or cyclodextrins, are threaded along an axle to give a pseudo[3]rotaxane architecture **V–VII** (Figure 2), where three sequential stereoisomers can arise. We showed that this stereoisomerism can be effectively controlled when two calix[6]arene wheels are threaded along a bis(benzylalkylammonium) axle [19], where the stereoselective formation of the pseudo[3]rotaxane with *endo*-alkyl orientation **VIII** was observed [19].

**Figure 3:**
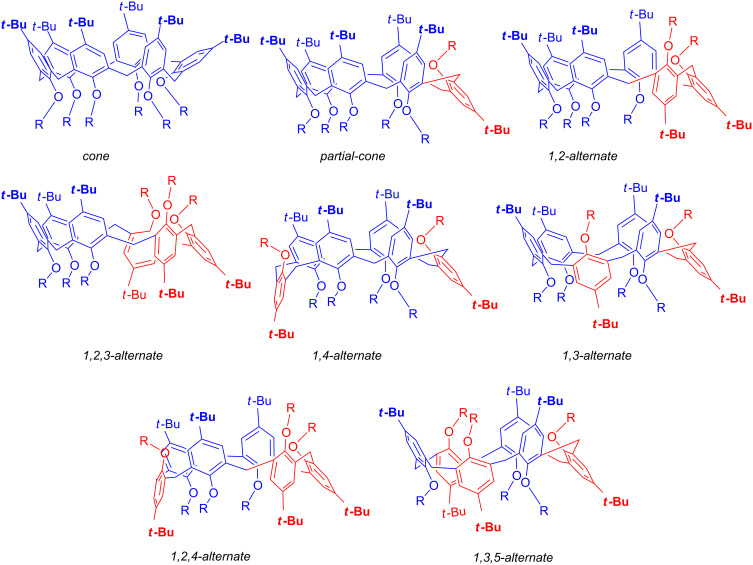
The possible 8 discrete conformations of a calix[6]arene macrocycle [26].

Calixarene macrocycles [22] have found numerous applications in several areas of supramolecular chemistry, such as (bio)molecular recognition [23] and catalysis [24]. The widespread use of the calixarene derivatives is due to their convenient synthesis and to their chemical and conformational versatility [25]. In fact, calixarene macrocycles present a conformational isomerism that in the case of calix[6]arenes gives rise to eight discrete conformations (Figure 3) [26]: *cone, partial-cone, 1,2-alternate, 1,3-alternate, 1,4-alternate, 1,2,3-alternate, 1,2,4-alternate,* and *1,3,5-alternate*. This conformational versatility has long attracted much attention, and therefore empirical rules have been reported in order to assign the calixarene conformations [27–28]. The “^1^H NMR Δδ” rule reported by Gutsche [29], is focused on the difference of chemical shifts between each pair of calixarene ArCH_2_Ar methylene protons. These can show diasterotopicity resulting in AX or AB systems. Specifically, a ^1^H NMR methylene proton Δδ value of at least 0.7 shows that the two respective proximal aromatic rings are oriented *syn*, as in the *cone* conformation. In contrast, a Δδ value of 0.3 or less is attributable to an *anti-*orientation between the phenol rings, as in alternate conformations. The de Mendoza’s “^13^C NMR single rule” [30–31], is focused on the ^13^C NMR chemical shift of the ArCH_2_Ar methylene C, which is 30–33 ppm for the *syn-*orientation of the proximal phenol rings and typically 36–39 ppm with *anti*-positioned phenol rings as in alternate conformations.

**Figure 4:**
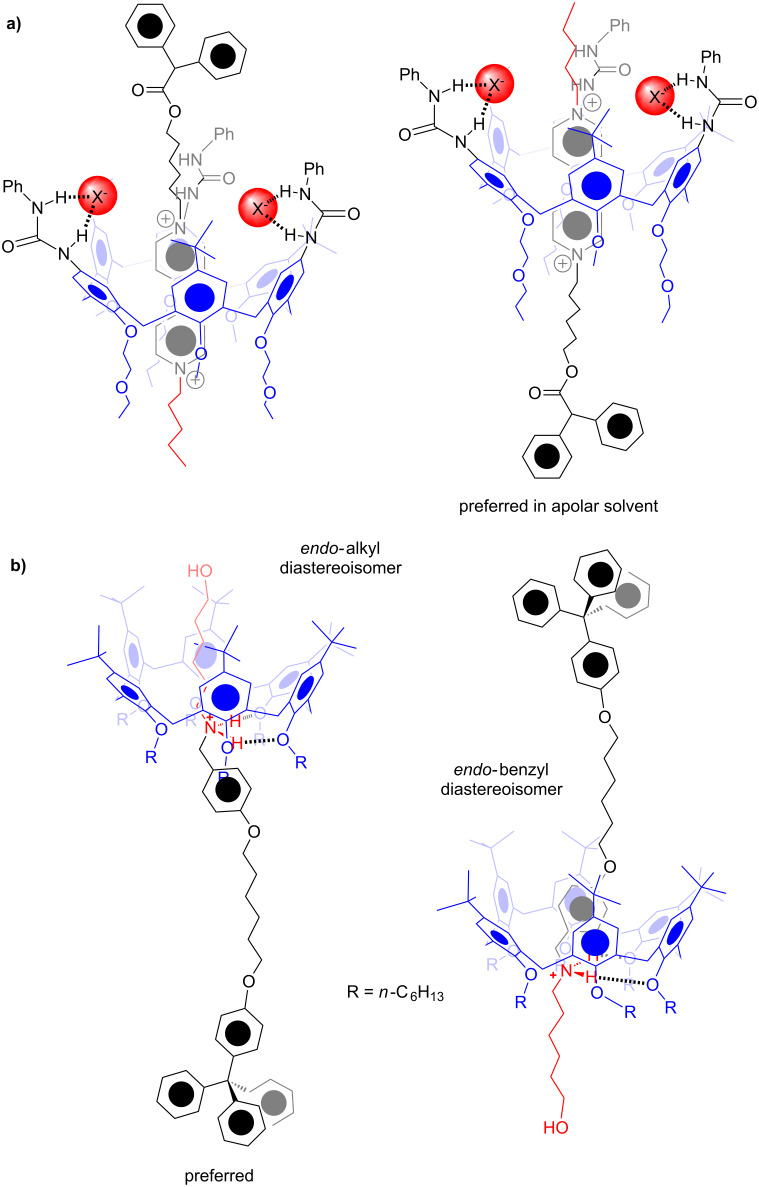
Diastereoisomeric pseudorotaxanes obtained by threading a directional calixarene wheel with directional axles.

As exemplified above, the calix[6]arene macrocycle has been widely used as wheel for the assembly of pseudorotaxane architectures [32–33], where it usually adopts a *cone* conformation. The examples reported by us [33–38] (Figure 4b) and by Arduini [32,39] (Figure 4a) showed that the directionality of the calixarene wheel in the *cone* conformation plays a pivotal role in the formation of stereoisomeric directional pseudo[2]rotaxanes, rotaxanes, and catenanes. Also in this case [38], we were able to obtain a stereoselective threading of the *cone* calix[6]arene-wheel with alkylbenzylammonium axles (Figure 4b), in which the *endo*-alkyl pseudo[2]rotaxane stereoisomer was the favoured one [38].

The threading of calix[6]arene macrocycles in conformations different than the cone one has been rarely observed [17]. Interestingly, the assembling of interpenetrated structures in which the wheel adopts different conformational isomers, could pave the way to mechanomolecules which exhibit novel isomeric forms.

Prompted by these considerations, some examples of pseudorotaxane isomers in which the isomerism arises by the different conformations adopted by the calixarene wheel are reported here.

## Results and Discussion

**Scheme 1:**
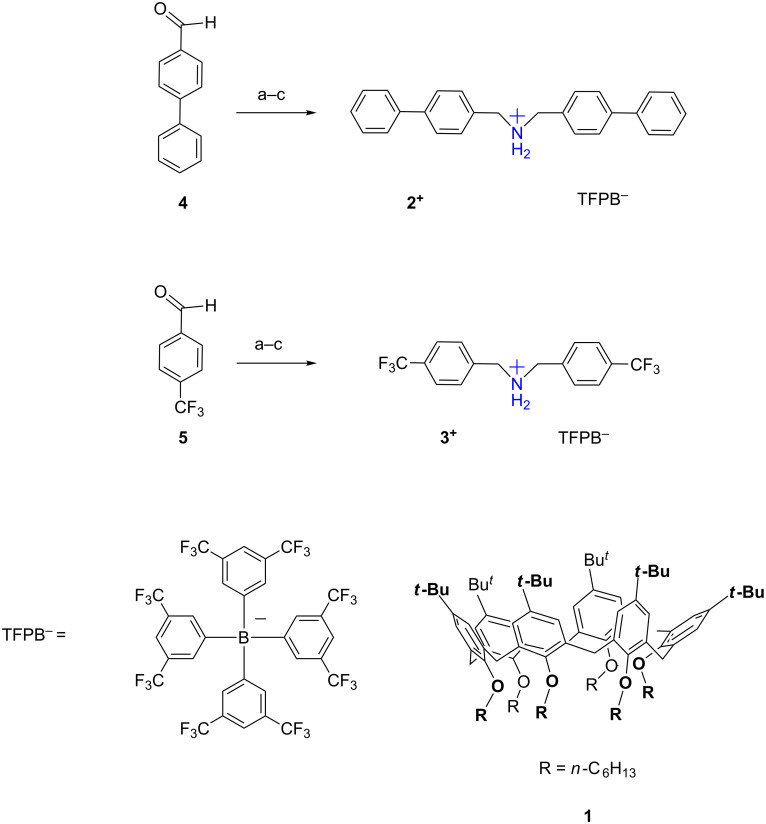
Synthesis of threads **2^+^** and **3^+^**. Reagents and conditions: a) hexamethyldisilazane, LiClO_4_, 30 min, 60 °C; b) CH_3_OH, NaBH_4_, 2 h, 25 °C; c) TFPBNa, dry MeOH, 25 °C, 18 h.

With this goal in mind, we conducted an initial screening in order to select the ammonium axles and the calix[6]arene-wheel most suitable for our purposes. At the end of our screening, we focused our attention on hexahexyloxycalix[6]arene **1** as the wheel and bis(4-biphenylmethyl)ammonium (**2^+^**) and bis(4-trifluoromethylbenzyl)ammonium (**3^+^**, TFPB^–^ salts) as the threads. The synthetic pathway to **2^+^**·TFPB^−^ and **3^+^**·TFPB^−^ salts is outlined in Scheme 1, while calix[6]arene **1** was obtained following a known procedure [40].

**Figure 5:**
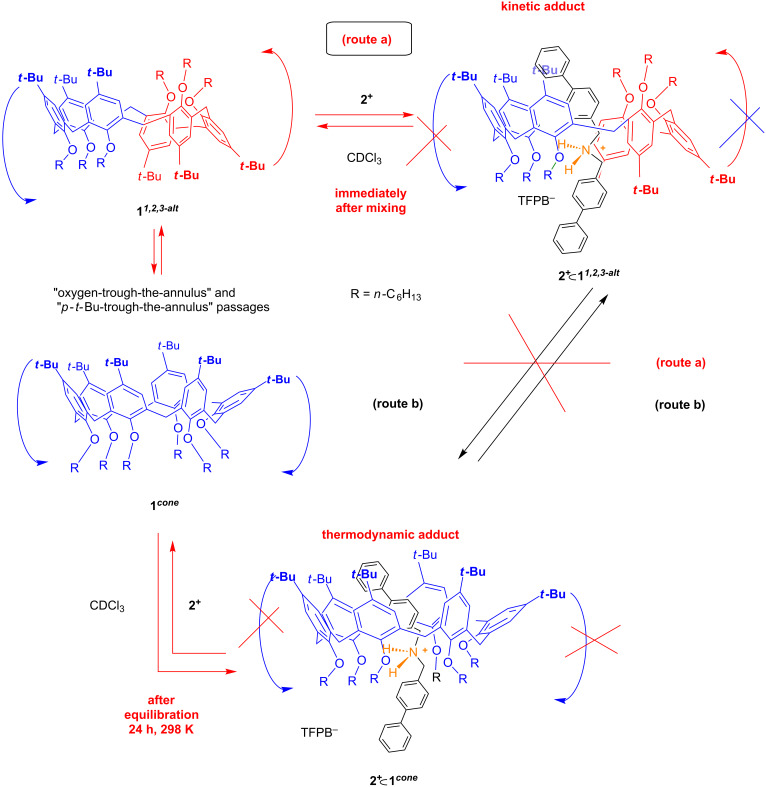
Possible mechanism for the formation of the two atropoisomeric pseudo[2]rotaxanes **2^+^**
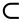
**1*^cone^*** and **2^+^**
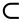
**1*^1,2,3-alt^***.

The ^1^H NMR spectrum of hexahexyloxycalix[6]arene **1** in CDCl_3_ at 298 K shows broad ArCH_2_Ar signals indicative of a conformational mobility of the macrocycle in which the inversion between the calix[6]arene conformations (Figure 5), occurs by means of rotation around the ArCH_2_Ar bonds.

By lowering the temperature, the ArCH_2_Ar signal decoalesced to form a single AX system (3.34/4.49 ppm) and one broad singlet (3.77 ppm). This pattern is only compatible with the presence of a *1,2,3-alternate* conformation of calix[6]arene **1** (Figure 5). This was confirmed by a 2D HSQC spectrum of **1** at 233 K which evidenced the presence of ArCH_2_Ar correlations between the AX system at 3.34/4.49 ppm with a carbon resonance at 29.4 ppm, related to *syn*-oriented Ar rings [29]. Diagnostic of the presence of the *1,2,3-alternate* conformation of **1** is the presence of the broad singlet at 3.71 ppm which correlates in the HSQC spectrum with a carbon resonance at 34.1 ppm [30], related to *anti*-oriented Ar rings. A close inspection of the 1D and 2D NMR spectrum of **1** in CDCl_3_ at 233 K clearly evidenced the presence of a less abundant conformer of **1**. The nature of this minor conformer can be inferred by the work of Reinhoudt and co-workers which showed [41] that the conformations preferentially adopted by calix[6]arene hexaethers are the *cone* and *1,2,3-alternate* ones. In accordance, 2D COSY and HSQC spectra of **1** at 233 K clarified that this minor conformer was the *cone* one through the presence of an AX system at 3.35/4.42 ppm (COSY), which correlates with a carbon resonance at 29.1 ppm (HSQC), related to *syn*-oriented Ar rings (*cone* conformation). The coalescence temperature of the methylene protons was ascertained at 328 K in CDCl_3_; below this temperature the conformations of **1** were frozen, while at temperatures above 328 K the conformational interconversion is fast with respect to the NMR time scale (400 MHz). From the coalescence data we calculated a barrier of 14.6 kcal/mol for this process. In summary, the VT ^1^H NMR studies indicate that the *1,2,3-alternate* is the most stable conformation for hexahexyloxycalix[6]arene **1** in solution. This conclusion is in perfect accord with the results previously reported by Reinhoudt [41], which evidenced an increased stabilization of the *1,2,3-alternate* conformation of calix[6]arenes when the alkyl substituents at the lower rim are increased in size [41].

**Figure 6:**
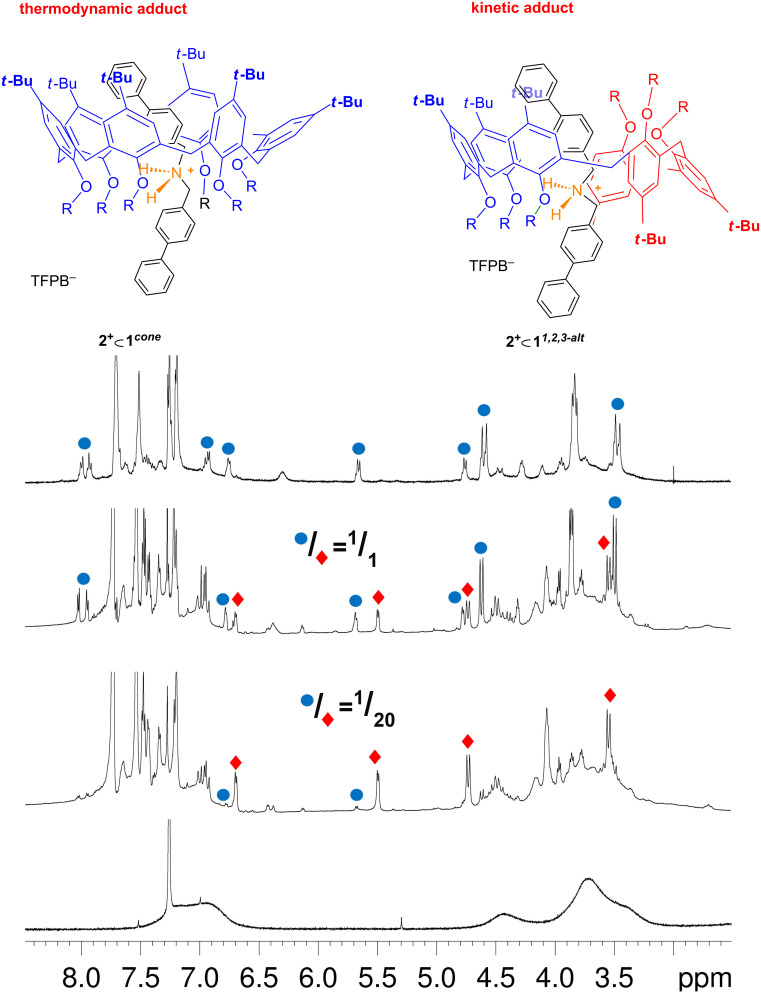
^1^H NMR spectra (600 MHz, CDCl_3_, 298 K) of, from bottom to top: hexahexyloxycalix[6]arene **1**; a 1:1 mixture (0.003 M) of **1** and **2^+^·TFPB^–^** immediately after mixing; after 10 h; after 18 h.

As expected [40], no evidence of interaction between **2^+^** and **1** was detected by NMR, when **2^+^** was added as its chloride salt to a CDCl_3_ solution of **1**. However, when **2^+^** was added as its TFPB^−^ salt to a CDCl_3_ solution of **1**, then dramatic changes were observed in the ^1^H NMR spectrum of **1** (Figure 6).

In detail, immediately after the mixing of **1** and **2^+^** we observed the sharpening of all signals and the appearance of an AX system at 5.50/6.70 ppm attributable to aromatic H-atoms of the axle **2^+^** shielded inside the calixarene cavity. These changes were indicative of the formation of a pseudorotaxane **2^+^**
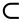
**1**. With this result in hand, we turned our attention to the conformation adopted by the calix[6]arene-wheel **1** in pseudorotaxane **2^+^**
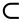
**1**. A 2D COSY spectrum of 1:1 mixture of **1** and **2^+^**, immediately after mixing in CDCl_3_, revealed the presence of a single AX systems at 3.53/4.73, which correlates with a carbon resonance at 28.9 ppm, respectively, due to the ArCH_2_Ar methylene groups between *syn-*oriented Ar rings. A close inspection of the 2D HSQC spectrum revealed the presence of a cross-peak at 3.93/36.5 ppm attributable to an ArCH_2_Ar methylene bridge between *anti*-oriented Ar rings. These data clearly indicate that calixarene-wheel **1** adopts the *1,2,3-alternate* conformation in pseudorotaxane **2^+^**
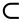
**1*^1,2,3-alt^*** (Figure 5 and Figure 6).

A further inspection of the 1D and 2D (COSY-45 and HSQC) spectra of the 1:1 mixture of **1** and **2^+^** in CDCl_3_ immediately after mixing, revealed the presence of a less abundant pseudo[2]rotaxane species in which probably the calixarene wheel **1** adopts a *cone* conformation **2^+^**
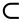
**1*^cone^*** (Figure 5). Initially, the ratio between the two isomeric pseudorotaxane **2^+^**
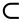
**1*^cone^***/**2^+^**
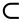
**1*^1,2,3-alt^*** is 1/20, as calculated by integration of the corresponding ^1^H NMR signals. Interestingly, after 10 h at 298 K (Figure 6), the intensity of the ^1^H NMR signals of pseudorotaxane **2^+^**
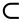
**1*^1,2,3-alt^*** was decreased while that of **2^+^**
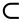
**1*^cone^*** was increased. After 18 h at 298 K, the disappearance of **2^+^**
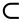
**1*^1,2,3-alt^*** was complete and only **2^+^**
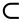
**1*^cone^*** pseudorotaxane could be detected by 1D and 2D NMR studies (Figure 6). In fact, a 2D COSY spectrum of the 1:1 mixture of **1** and **2^+^** in CDCl_3_, after 18 h at 298 K, showed the presence of an ArCH_2_Ar AX system at 3.47/4.62 ppm which correlates in the HSQC spectrum with a carbon resonance at 28.4 ppm related to *syn-*oriented Ar rings. An AX system was present in the COSY spectrum at 4.78/5.68 ppm attributable to aromatic protons of the axle **2^+^** shielded inside the calixarene cavity. This shielded AX system correlates in the HSQC spectrum with aromatic carbon resonances at 129.8 and 126.8 ppm, respectively.

The ^1^H NMR spectrum of the mixture of **1** and **2^+^** in CDCl_3_ remained unchanged after 48 h at 298 K, thus showing that the system had reached the equilibrium condition. At this point, an apparent association constant of 6.2 ± 0.3 × 10^3^ M^–1^ was calculated by quantitative ^1^H NMR analysis (tetrachloroethane as internal standard) [37] for the formation of **2^+^**
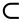
**1*^cone^*** pseudorotaxane. In conclusion, after the initial formation of the kinetically favored pseudorotaxane **2^+^**
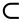
**1*^1,2,3-alt^*** (Figure 5), the thermodynamic pseudorotaxane **2^+^**
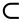
**1*^cone^*** prevails (Figure 5 and Figure 6). As demonstrated above, the *1,2,3-alternate* conformation of **1** is the most populated in solution, consequently, the threading of this conformation, besides being faster, it is also favored by its abundance in solution.

**Figure 7:**
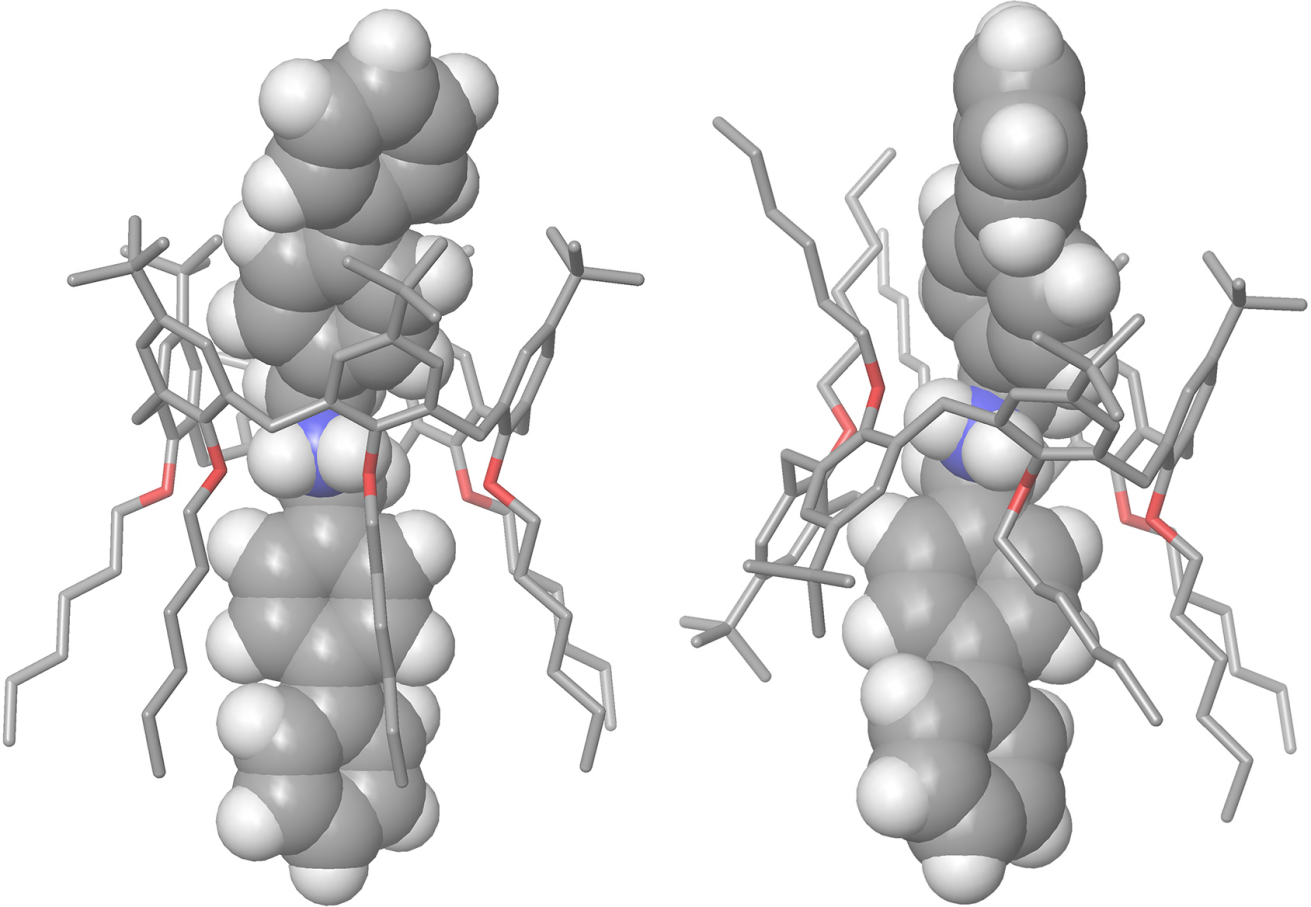
DFT-optimized structures of the: (left) **2^+^**
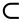
**1*^cone^*** and (right) **2^+^**
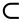
**1*^1,2,3-alt^*** pseudorotaxane atropoisomers calculated at B3LYP/6-31G(d,p) level of theory and using Grimme’s dispersion corrections (IOp(3/124 = 3)).

The greater thermodynamic stability of the **2^+^**
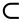
**1*^cone^*** atropoisomer over the **2^+^**
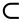
**1*^1,2,3-alt^*** one, was confirmed by DFT calculations at the B3LYP/6-31G(d,p) level of theory using Grimme’s dispersion corrections (IOp(3/124=3)) [42]. The DFT-optimized structure of the **2^+^**
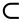
**1*^cone^*** atropoisomeric pseudorotaxane (Figure 7, left) results stabilized by two H-bond interactions between the ammonium group and the oxygen atoms of the calixarene wheel **1**, (average N···O distance = 3.10 Å; average N–H···O angle = 157°). In addition, C–H···π interactions were detected among the methylene groups of the axle **2^+^** inside the calix cavity, and the aromatic rings of **1** [42], ( average C–H···π*^centroid^* distance = 3.17 Å [42]; average C–H···π*^centroid^* angle = 160° [43]).

In addition, the biphenyl portion of **2^+^** hosted inside the calix cavity was involved in π···π interactions with the aromatic walls (Figures S11–S13, Supporting Information File 1) and C–H···π interactions with the *tert*-butyl groups of the calixarene wheel (Figure S13, Supporting Information File 1). Differently, in the DFT-optimized structure of **2^+^**
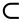
**1*^1,2,3-alt^*** atropoisomer (Figure 7, right), the stabilization of the **2^+^**
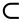
**1*^1,2,3-alt^*** atropoisomer was brought, principally by two H-bonding interactions between the ammonium group of **2^+^** and the oxygen atoms of *anti*-oriented phenol rings of **1** with an average N···O distance of 3.05 Å and a narrower N–H···O angle of 167.1°. Single-point calculations at the B3LYP/6-31G(d,p) level of theory using Grimme’s dispersion corrections (IOp(3/124=3)), indicated that the **2^+^**
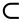
**1*^cone^*** atropoisomer was more stable than the **2^+^**
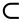
**1*^1,2,3-alt^*** one by 2.4 kcal mol^−1^. At this point, it is worthy to consider the interconversion between the two isomeric pseudorotaxane **2^+^**
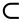
**1*^1,2,3-alt^*** and **2^+^**
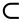
**1*^cone^***. It could take place through two possible mechanisms (Figure 5): a) de-threading of axle **2^+^** from **2^+^**
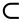
**1*^1,2,3-alt^*** and a subsequent re-threading with **1** in a *cone* conformation; b) a direct conformational interconversion between the *1,2,3-alternate* and *cone* conformations of the calixarene wheel **1** in both **2^+^**
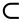
**1** pseudorotaxanes. Previously reported data [34] clearly showed that the mechanism “b” in Figure 5 can be ruled out because the presence of an axle inside the cavity of **1** impedes the "through-the-annulus" passage of both rims of **1**. From this consideration, we concluded that the two pseudorotaxanes **2^+^**
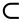
**1*^1,2,3-alt^*** and **2^+^**
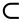
**1*^cone^*** can be considered as two atropoisomeric forms. In fact, the interconversion between them cannot be obtained by simple rotation around chemical bonds of the calixarene wheel, which is blocked by the presence of the axle inside its cavity.

**Figure 8:**
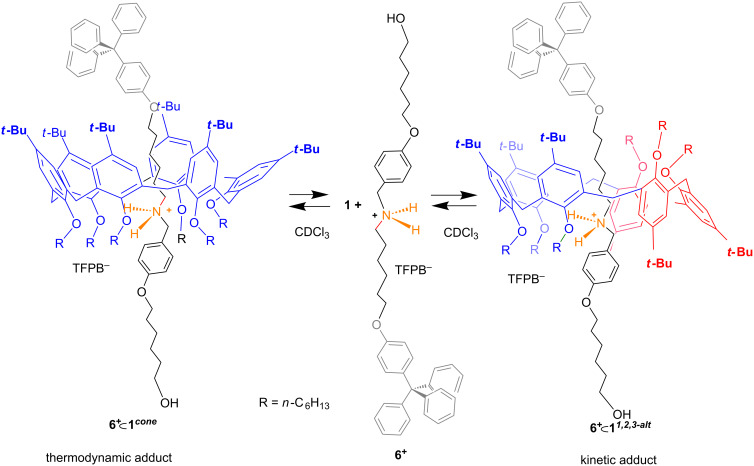
The two pseudorotaxane atropoisomers obtained by threading hexahexyloxycalix[6]arene **1** with monostoppered alkylbenzylammonium axle **6^+^**.

**Figure 9:**
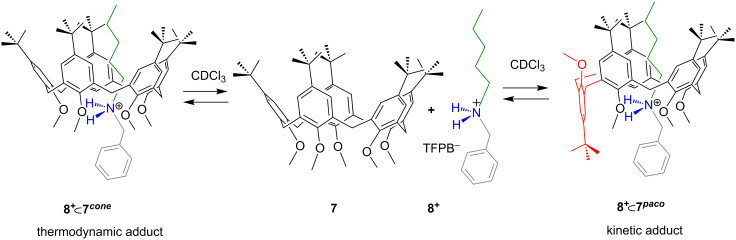
The two pseudorotaxane atropoisomers obtained by threading penta-*O*-methyl-*p-tert*-butylcalix[5]arene **7** with pentylbenzylammonium axle **8^+^**.

Previously [34] we reported a similar case in which the monostoppered alkylbenzylammonium axle **6^+^** gives rise to two atropoisomeric pseudorotaxanes **6^+^**
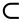
**1*^cone^*** and **6^+^**
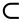
**1*^1,2,3-alt^*** (Figure 8). Also in this instance, the pseudorotaxanes **6^+^**
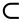
**1*^1,2,3-alt^*** and **6^+^**
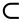
**1*^cone^*** were observed as the kinetic and thermodynamic adduct, respectively, with an interconversion time of 12 h at 353 K. A further example regards the threading of the narrower penta-*O*-methyl-*p-tert*-butylcalix[5]arene **7** with pentylbenzylammonium axle **8^+^** [35]. Two atropoisomeric pseudorotaxanes were formed, namely **8^+^**
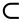
**7*^cone^*** and **8^+^**
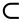
**7*^paco^*** (Figure 9), in which the calix[5]-wheel adopted a *cone* and a *partial-cone* conformation, respectively [35]. Also in this case, the atropoisomer with an “inverted” calixarene wheel **8^+^**
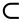
**7*^paco^*** is the kinetic product, while the other with a calix-*cone* conformation **8^+^**
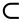
**7*^cone^*** is the thermodynamic one [35].

**Figure 10:**
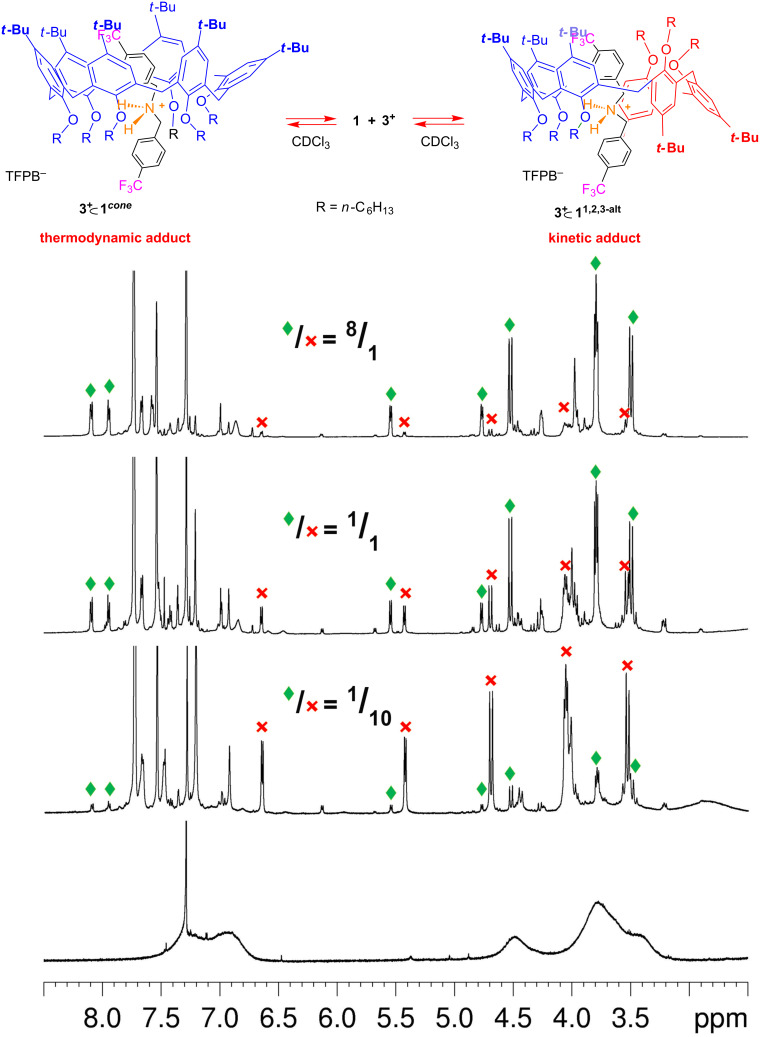
^1^H NMR spectra (600 MHz, CDCl_3_, 298 K) of, from bottom to top: hexahexyloxycalix[6]arene **1**; a 1:1 mixture (0.003 M) of **1** and **3^+^**·TFPB^−^ immediately after mixing; after 2 h; after 18 h, mechanism for the formation of the two atropoisomeric pseudo[2]rotaxanes **3^+^**
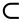
**1*^cone^*** and **3^+^**
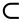
**1*^1,2,3-alt^***.

At this point we turned our attention to the threading properties of bis(4-trifluoromethylbenzyl)ammonium axle **3**^+^. When **1** and **3^+^**·TFPB^−^ were mixed in CDCl_3_ two atropoisomeric pseudo[2]rotaxane, **3^+^**
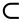
**1*^cone^*** and **3^+^**
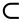
**1*^1,2,3-alt^*** (Figure 10), were formed in a 1/10 ratio, as revealed by 1D and 2D NMR studies. Also in this case, after equilibration at 298 K for 24 h, this preference was reversed in favour of the **3+**
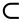
**1*^cone^*** atropoisomer, with a **3^+^**
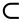
**1*^cone^***/**3^+^**
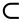
**1*^1,2,3-alt^*** ratio of 8/1. From the equilibrium mixture, an apparent association constant of 9.3 ± 0.4 × 10^2^ M^−1^ was calculated by quantitative ^1^H NMR analysis (tetrachloroethane as internal standard) for the formation of **3^+^**
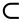
**1*^cone^*** pseudorotaxane. In a similar way, an apparent association constant of 120 ± 15 M^−1^ was found for **3^+^**
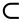
**1*^1,2,3-alt^*** pseudorotaxane.

As evidenced for axle **2^+^**, also in this case, after the initial formation of the kinetic pseudorotaxane **3^+^**
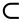
**1*^1,2,3-alt^*** (Figure 10), the thermodynamic atropoisomer **3^+^**
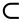
**1*^cone^*** prevails. However, differently from the **2^+^** case where the kinetic product was no longer detectable in the final equilibrium mixture, here a sizeable amount of the kinetic pseudorotaxane **3^+^**
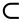
**1*^1,2,3-alt^*** can be observed at the equilibrium indicating a smaller energy difference with respect to the thermodynamic atropoisomer **3^+^**
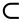
**1*^cone^***. This can be ascribed to a higher destabilization of the *cone* atropoisomer due to a higher number of unfavourable “fluorophobic” interactions between the CF_3_ group and the *t*-Bu-Ar moieties.

## Conclusion

We have here reported a study on isomeric pseudorotaxanes in which the isomerism arises by the different conformation adopted by the calix[6]arene wheel. Among the eight possible discrete conformations of the calix[6]arene macrocycle, the *cone* and *1,2,3-alternate* ones were observed in the pseudorotaxane architectures obtained by threading a hexahexyloxycalix[6]arene with axles bearing biphenyl or trifluoromethylbenzyl moieties. The interconversion between the *cone* and *1,2,3-alternate* conformations occurs, in free calix[6]arene, by means of the “oxygen-through-the-annulus” and/or “*p*-substituent-through-the-annulus” passages. The presence of the ammonium axles inside the calixarene cavity prevents these passages; consequently two atropoisomeric pseudorotaxanes were formed. We showed that the interconversion between the two atropoisomeric pseudorotaxanes can only occur through a mechanism of de-threading/re-threading of the axle. In all the examined cases, the *1,2,3-alternate* and *cone* atropoisomers are the kinetic and thermodynamic pseudorotaxane, respectively. We do believe that novel and intriguing calixarene-based mechanomolecules, with expanded properties or functions, could be obtained by an appropriate stoppering or catenation of such atropoisomeric pseudorotaxanes.

## Experimental

ESI(+)–MS measurements were performed on a Micromass Bio-Q triple quadrupole mass spectrometer equipped with electrospray ion source, using a mixture of H_2_O/CH_3_CN (1:1) and 5% HCOOH as solvent. Flash chromatography was performed on Merck silica gel (60, 40–63 μm). All chemicals were reagent grade and were used without further purification. Anhydrous solvents were purchased from Aldrich. When necessary compounds were dried in vacuo over CaCl_2_. Reaction temperatures were measured externally. Reactions were monitored by TLC on Merck silica gel plates (0.25 mm) and visualized by UV light, or by spraying with H_2_SO_4_–Ce(SO_4_)_2_ or phosphomolybdic acid. NMR spectra were recorded on a Bruker Avance-600 spectrometer [600 (^1^H) and 150 MHz (^13^C)], Bruker Avance-400 spectrometer [400 (^1^H) and 100 MHz (^13^C)], Bruker Avance-300 spectrometer [300 (^1^H) and 75 MHz (^13^C)], or Bruker Avance-250 spectrometer [250 (^1^H) and 63 MHz (^13^C)]; chemical shifts are reported relative to the residual solvent peak (CHCl_3_: δ 7.26, CDCl_3_: δ 77.23; CD_3_OH: δ 4.87, CD_3_OD: δ 49.0;). Standard pulse programs, provided by the manufacturer, were used for 2D COSY-45, 2D ROESY and 2D NOESY/EXSY experiments.

### General procedure for the preparation of **2^+^** and **3^+^**·TFPB^−^ salts

Derivative **4** (or **5**, 2.2 mmol) was dissolved at 60 °C in liquid (Me_3_Si)_2_NH (0.71 g, 4.4 mmol, 0.92 mL), LiClO_4_ (0.02 g, 2.2 mmol) was added and the reaction was kept under stirring at 60 °C until a white solid was formed (30 min). The solution was allowed to cool down at room temperature and dry MeOH (4.0 mL) was added. The mixture was kept under stirring for 2 h and then cooled at 0 °C. NaBH_4_ (1.12 g, 11.0 mmol) was added and the mixture was kept under stirring at 0 °C for 15 min and then allowed to warm up at room temperature. After 2 hours the solvent was removed, the solid was dissolved in ethyl acetate (100 mL) and washed with an aqueous saturated solution of NaHCO_3_ (100 mL) and H_2_O (50 mL). The organic layer was dried over Na_2_SO_4_ and the solvent was removed under reduced pressure, to give secondary amine derivative. Amine was used without further purification in the next step. Secondary amine derivative (1.16 mmol) was dissolved in MeOH (20 mL) at room temperature and an aqueous solution of HCl (37% w/w, 0.20 mL) was added dropwise. The mixture was kept under stirring for 30 min, until the formation of a white precipitate. The solid was collected by filtration, washed with MeOH (10 mL) and CH_3_CN (10 mL) and dried under vacuum to give the ammonium chloride derivative. The chloride salt (0.68 mmol) and sodium tetrakis[3,5-bis(trifluoromethyl)phenyl]borate (0.60 g, 0.68 mmol) were dissolved in dry MeOH (15 mL). The solution was stirred for 18 h in the dark, then the solvent was removed and deionized water was added, obtaining a light brown precipitate, that was filtered off and dried under vacuum to give threads **2**^+^ or **3^+^**.

#### Derivative **2^+^**

Light brown solid, 0.73 g, 0.60 mmol, 88% yield (respect chloride salt); mp 135–138 °C; ESI(+) MS (*m*/*z*): 350.2 (M^+^); ^1^H NMR (400 MHz, CD_3_OD, 298 K) δ 4.34 (s, 4H), 7.37–7.41 (overlapped, 6H), 7.61–7.67 (overlapped, 20H), 7.76 (d, *J* = 7.8 Hz, 4H); ^13^C NMR (100 MHz, CD_3_OD, 298 K) δ 51.7, 118.4, 118.4, 118.5, 121.7, 124.4, 127.1, 128.0, 128.8, 129.0, 130.0, 130.1, 130.2, 130.3(2), 130.5(2), 130.6, 131.2, 131.5, 135.8, 141.3, 143.9, 162.1, 162.6, 163.1, 163.6; anal. calcd for C_58_H_36_BF_24_N: C, 57.40; H, 2.99; found: C, 57.39; H, 3.01.

#### Derivative **3^+^**

Light brown solid, 0.57 g, 0.48 mmol, 70 % yield (respect chloride salt); mp 125–128 °C; ESI(+) MS (*m*/*z*): 334.1 (M^+^); ^1^H NMR (300 MHz, CD_3_OD, 298 K) δ 3.7 (s, 4H), 7.30–7.32 (overlapped, 20H); ^13^C NMR (75 MHz, CD_3_OD, 298 K) δ 52.6, 118.3, 118.5, 118.6, 122.0, 125.4, 127.3, 128.3, 128.9, 129.1, 130.0, 130.3, 130.4, 130.5(2), 130.6, 131.2, 131.6, 135.8, 141.4, 144.0, 162.2, 162.7, 163.2, 163.5; anal. calcd for C_48_H_26_BF_30_N: C, 48.14; H, 2.19; found: C, 48.13; H, 2.17.

### General procedure for the preparation of pseudorotaxane derivatives

The calixarene derivative **1** (3.0 mM) and ammonium salt **2^+^** or **3^+^** (3.0 mM) were dissolved in CDCl_3_ (0.5 mL). Each solution was sonicated for 15 min at room temperature and then was transferred into a NMR tube for 1D and 2D NMR spectra acquisition.

**Determination of apparent *K*_ass_ value for pseudorotaxanes 2^+^**
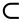
**1*^cone^*, 3^+^**
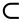
**1*^cone^* and 3^+^**
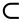
**1*^1,2,3-alt^*, by quantitative ^1^H NMR analysis.** The sample was prepared by dissolving calixarene **1** (3.0 × 10^−3^ M) and the ammonium TFPB salt **2^+^** or **3^+^** (3.0 × 10^−3^ M) in CDCl_3_ (0.5 mL) containing 1.0 μL of TCHE (*d* = 1.596 g/mL) as an internal standard. The complex concentration [complex] was evaluated by integration of the ^1^H NMR signal of TCHE versus the signals of the pseudorotaxane. The following equation was used to obtain the moles of the complex:


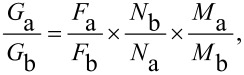


where *G*_a_ = grams of TCHE, *G*_b_ = grams of pseudorotaxane, *F*_a_ and *F*_b_ = areas of the signal of the TCHE and shielded aromatic protons of axle inside the calixarene cavity, *N*_a_ and *N*_b_ = numbers of nuclei that cause the signals (*N*_a_ for TCHE; *N*_b_ for pseudorotaxane) and *M*_a_ and *M*_b_ = molecular masses of TCHE (a) and pseudorotaxane (b).

## Supporting Information

File 1 VT NMR studies of hexyloxycalix[6]arene **1**, 2D COSY and HSQC spectra of atropoisomeric pseudorotaxanes, details of DFT calculations and atomic coordinates.
