# Supplementary material for: Calix[6]arene-based atropoisomeric pseudo[2]rotaxanes
Source: Beilstein J Org Chem. 2018 Aug 14;14:2112–24. doi: 10.3762/bjoc.14.186 (PMC6122310; doi:10.3762/bjoc.14.186)
Supplement: File 1 — VT NMR studies of hexyloxycalix[6]arene 1, 2D COSY and HSQC spectra of atropoisomeric pseudorotaxanes, details of DFT calculations and atomic coordinates. [file Beilstein_J_Org_Chem-14-2112-s001.pdf]

Supporting Information

for

Calix[6]arene-based atropoisomeric pseudo[2]rotaxanes

Carmine Gaeta, Carmen Talotta* and Placido Neri

Address: Dipartimento di Chimica e Biologia " A. Zambelli", Università di Salerno, Via Giovanni Paolo II 132, 84084 Fisciano (Salerno), Italy.

Email: Full name - email address of corresponding author

*Corresponding author

VT NMR studies of hexyloxycalix[6]arene 1, 2D COSY and HSQC spectra of atropoisomeric pseudorotaxanes, details of DFT calculations and atomic coordinates

Variable Temperature ^{1}H NMR Spectra of 1 ($CDCl_3$, 600 MHz)

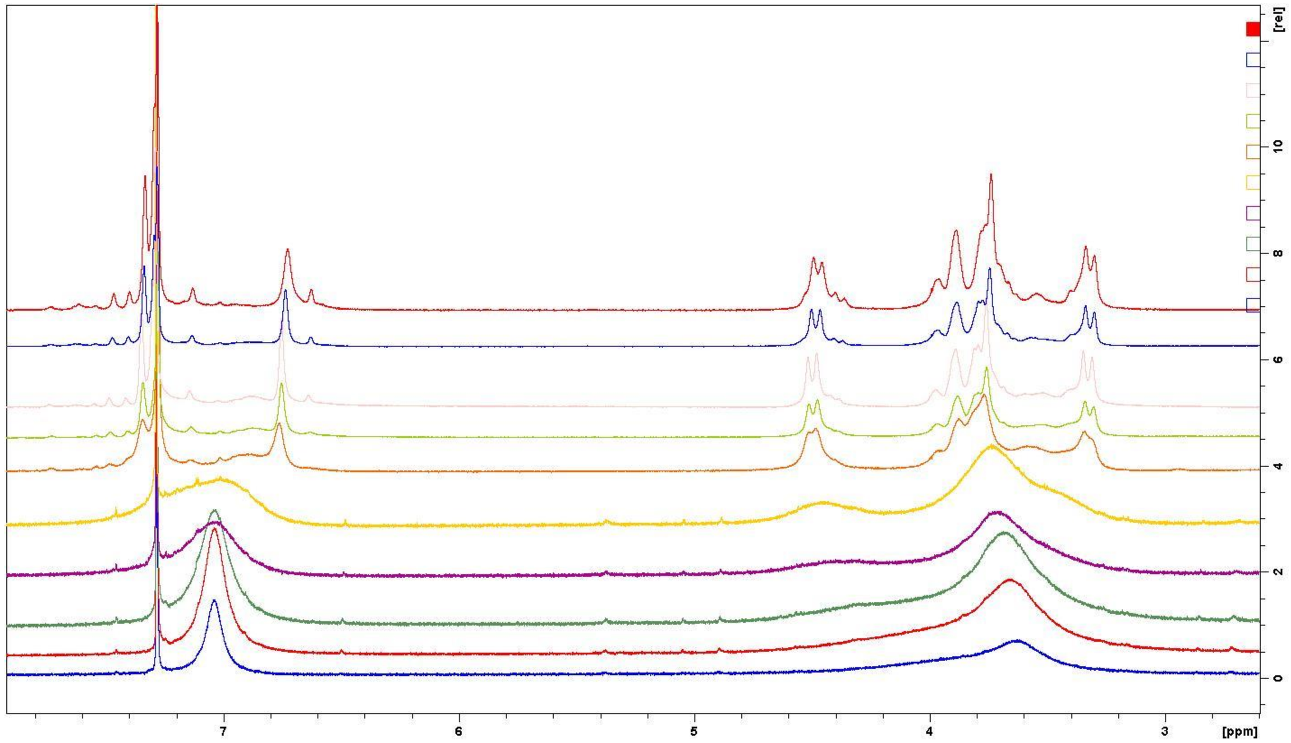

Figure S1: ^{1}H NMR spectra of **1** (400 MHz, $CDCl_3$) at (from bottom to top): **328** (Tc); 323; 318; 313; 298; 273; 263; 253; 243; 233 K.

$$\Delta G_c^{\neq} = aT_c\left[9.972 + \log\left(\frac{T_c}{\sqrt{\Delta\nu^2 + 6J^2}}\right)\right]$$

Kurland, R. J.; Rubin, M. B.; Wise, M. B. *J. Chem. Phys.* **1964**, *40*, 2426

T_C = 328 K; Δv = 466 Hz ; a = $4.575\cdot10^{-3}$ ($\Delta G^{\neq}_c$ in Kcal/mol)

2D Spectra of 1 at 233 K ($CDCl_3$, 600 MHz)

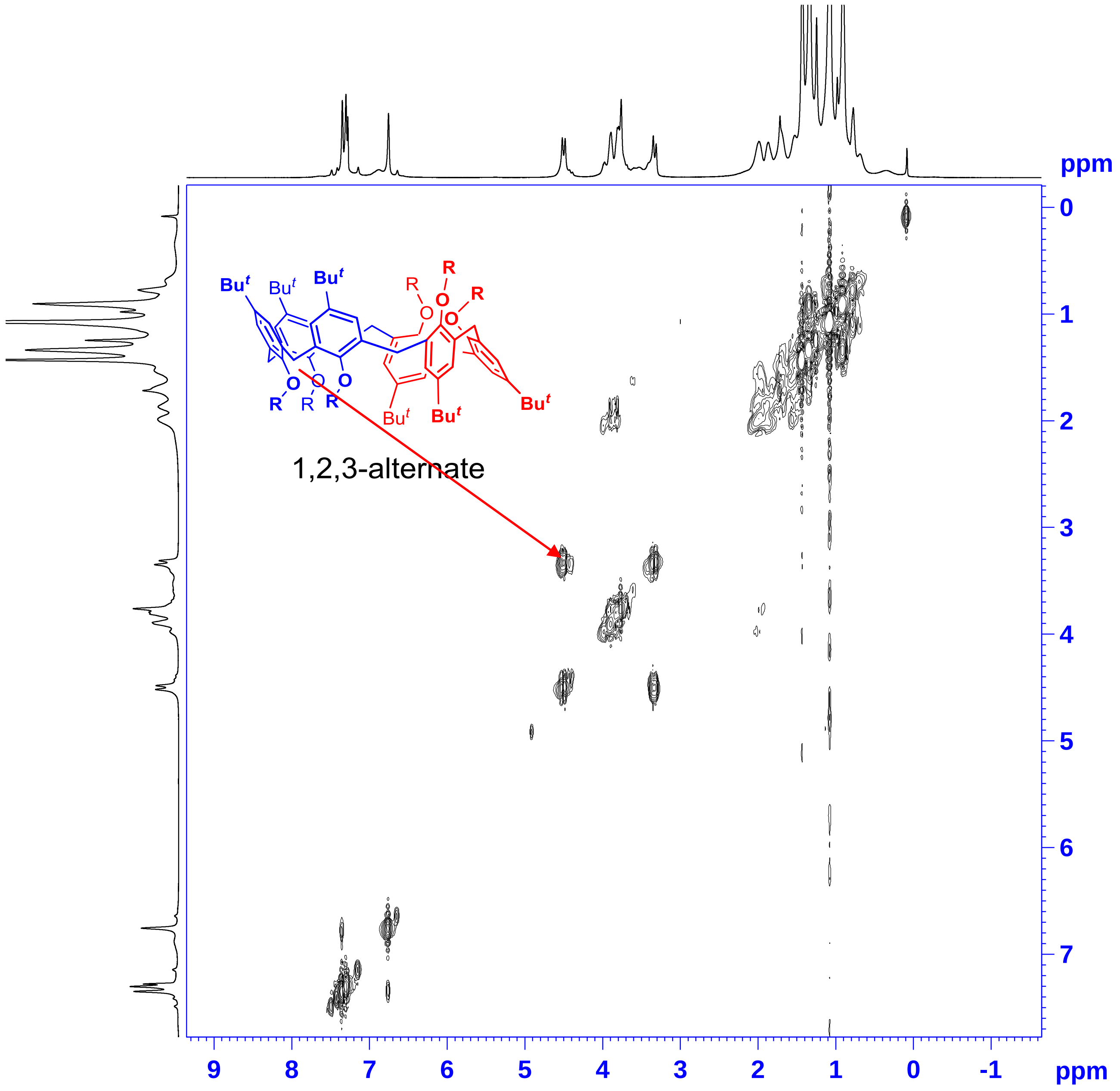

Figure S2: COSY spectrum of **1** (600 MHz, $CDCl_3$) at 233 K.

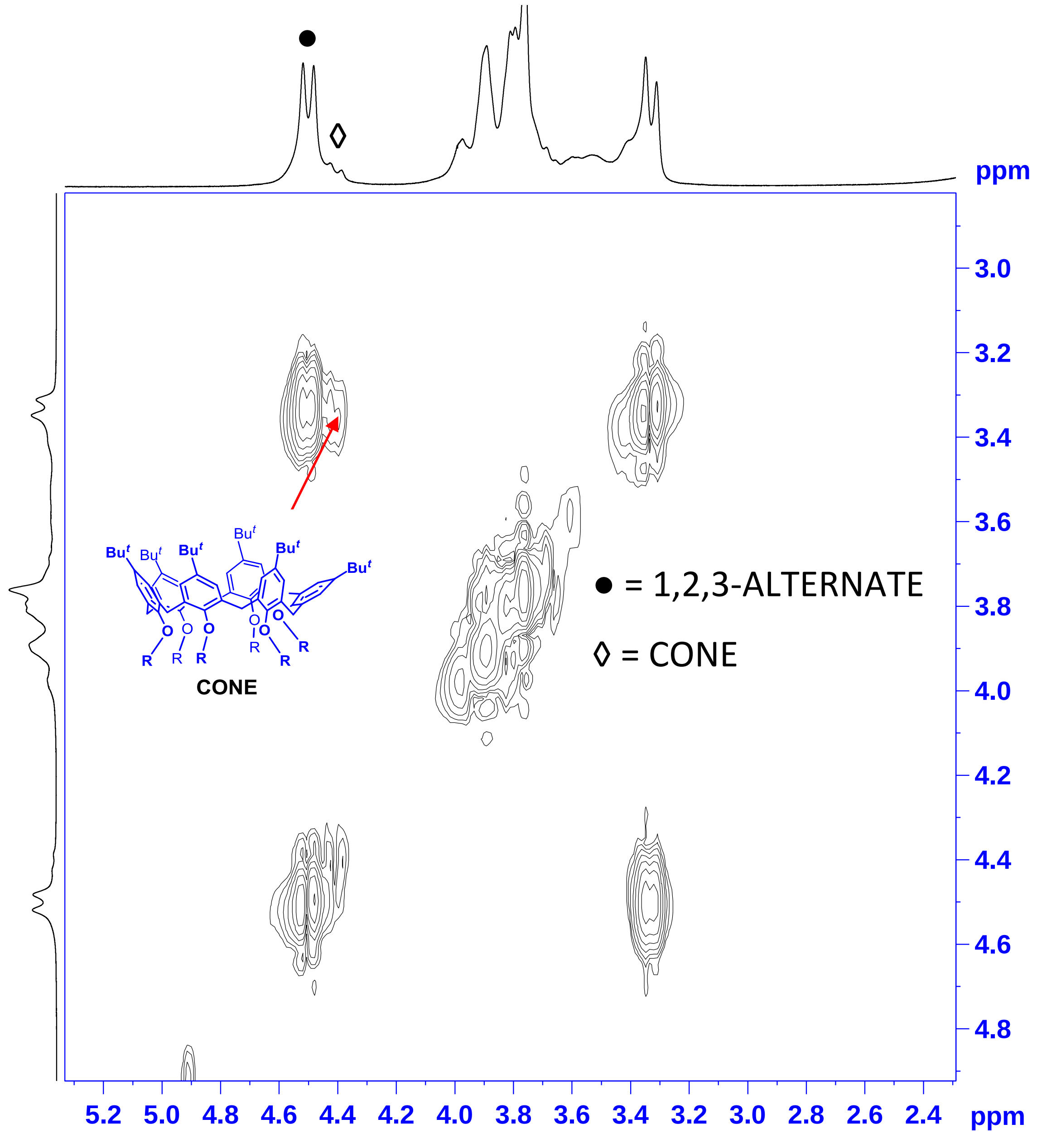

Figure S3: Expansion of the COSY spectrum of **1** (600 MHz, $CDCl_3$) at 233 K.

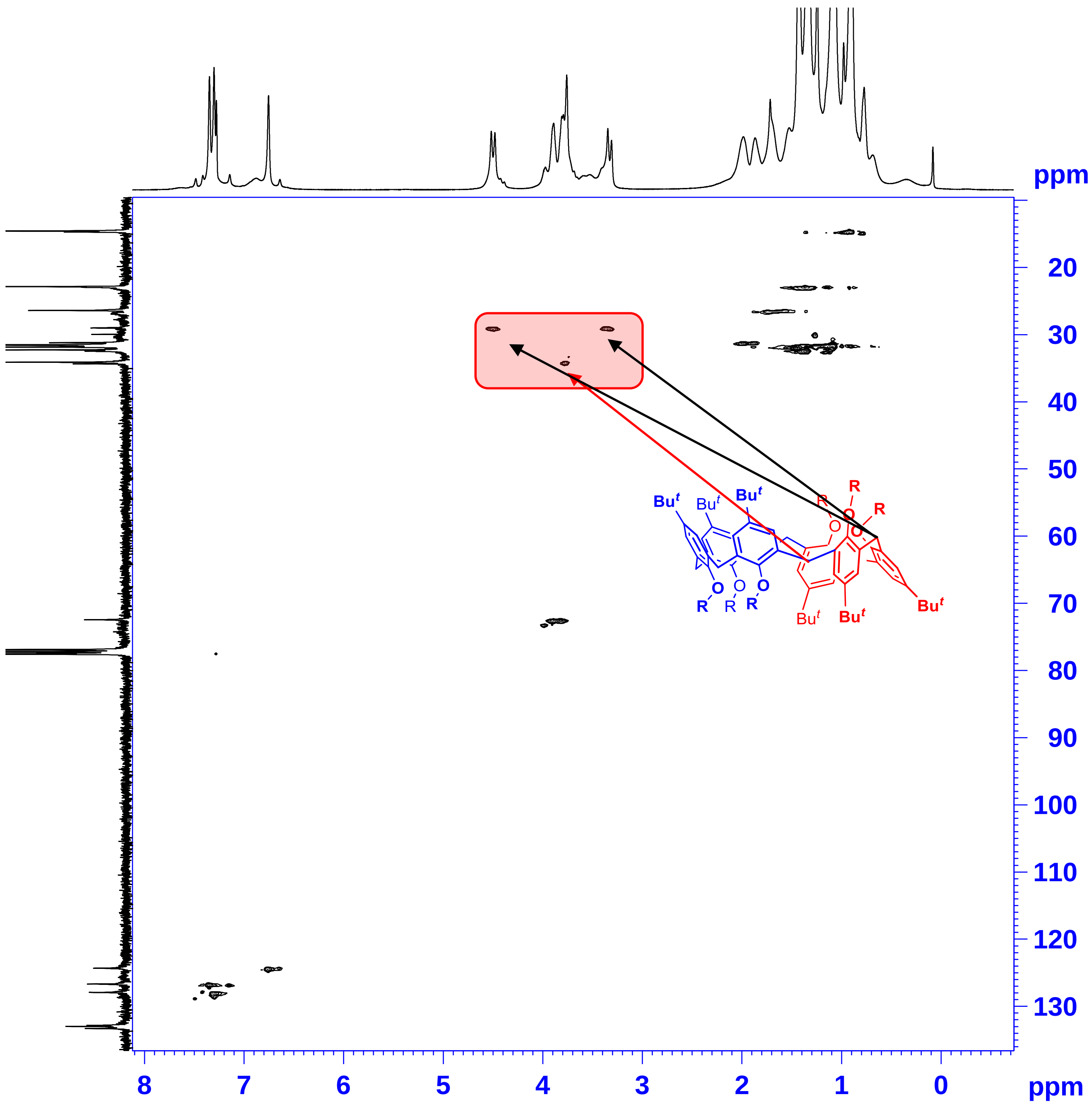

Figure S4: HSQC spectrum of **1** (600 MHz, $CDCl_3$) at 233 K.

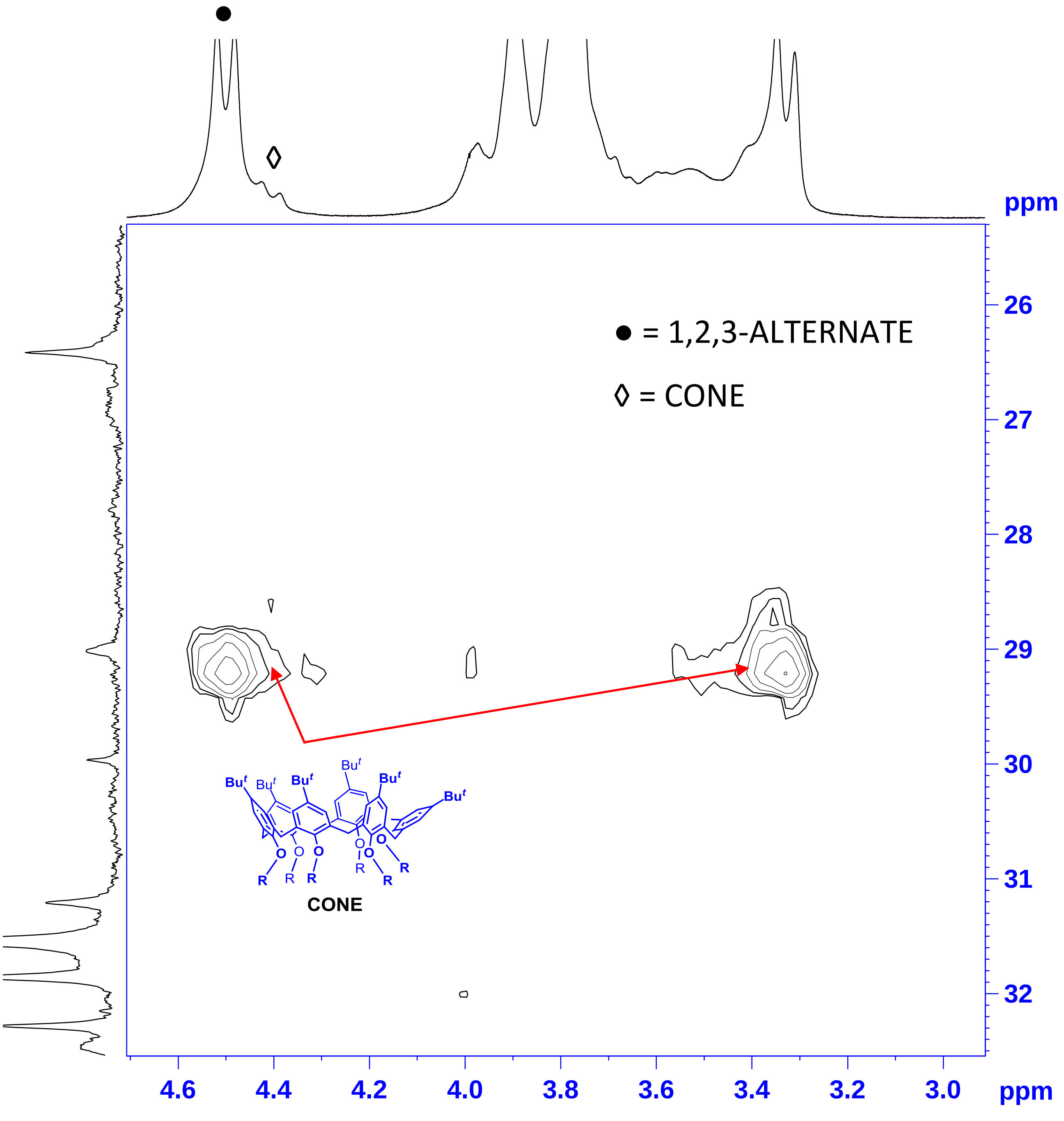

Figure S5: Expansion of the HSQC spectrum of **1** (600 MHz, $CDCl_3$) at 233 K.

2D COSY spectrum of $2^+\subset 1^{cone}$ and $2^+\subset 1^{1,2,3\text{-}alt}$ atropoisomers at 298 K (CDCl$_3$, 600 MHz)

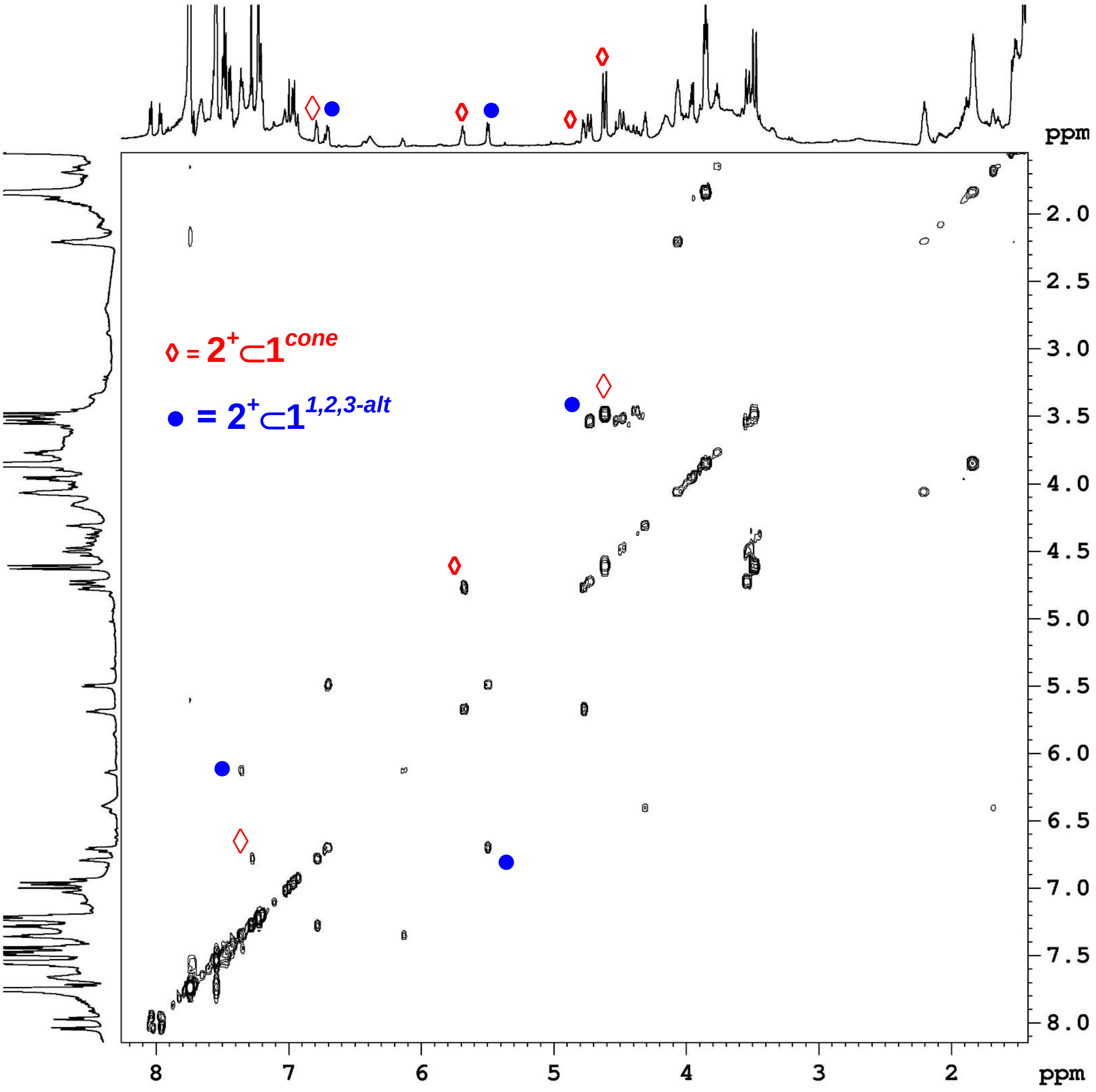

Figure S6: COSY spectrum (600 MHz, CDCl$_3$, 298 K) of a 1:1 mixture (0.003 M) of **1** and **2^+·TFPB$^-$** after 10 h by mixing.

2D COSY spectrum of $2^{+}\subset1^{cone}$ atropoisomer at 298 K ($CDCl_3$, 600 MHz)

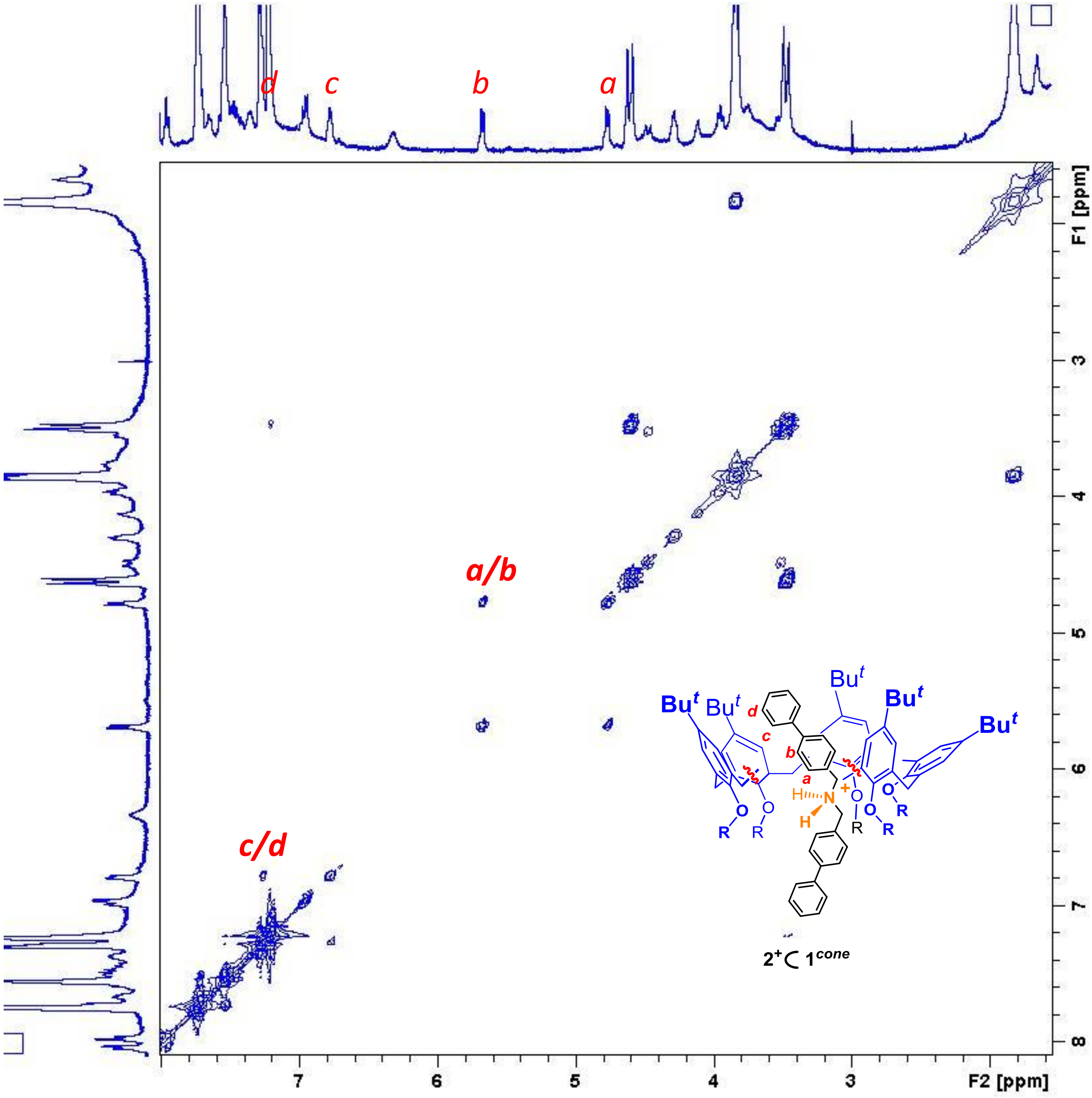

Figure S7: COSY spectrum (600 MHz, $CDCl_3$, 298 K) of a 1:1 mixture (0.003 M) of **1** and $\mathbf{2^{+}\cdot TFPB^{-}}$ after 18 h by mixing, $\mathbf{2^{+}\subset1^{cone}}$.

2D HSQC spectrum of $2^{+}\subset 1^{cone}$ atropoisomer at 298 K ($CDCl_3$, 600 MHz)

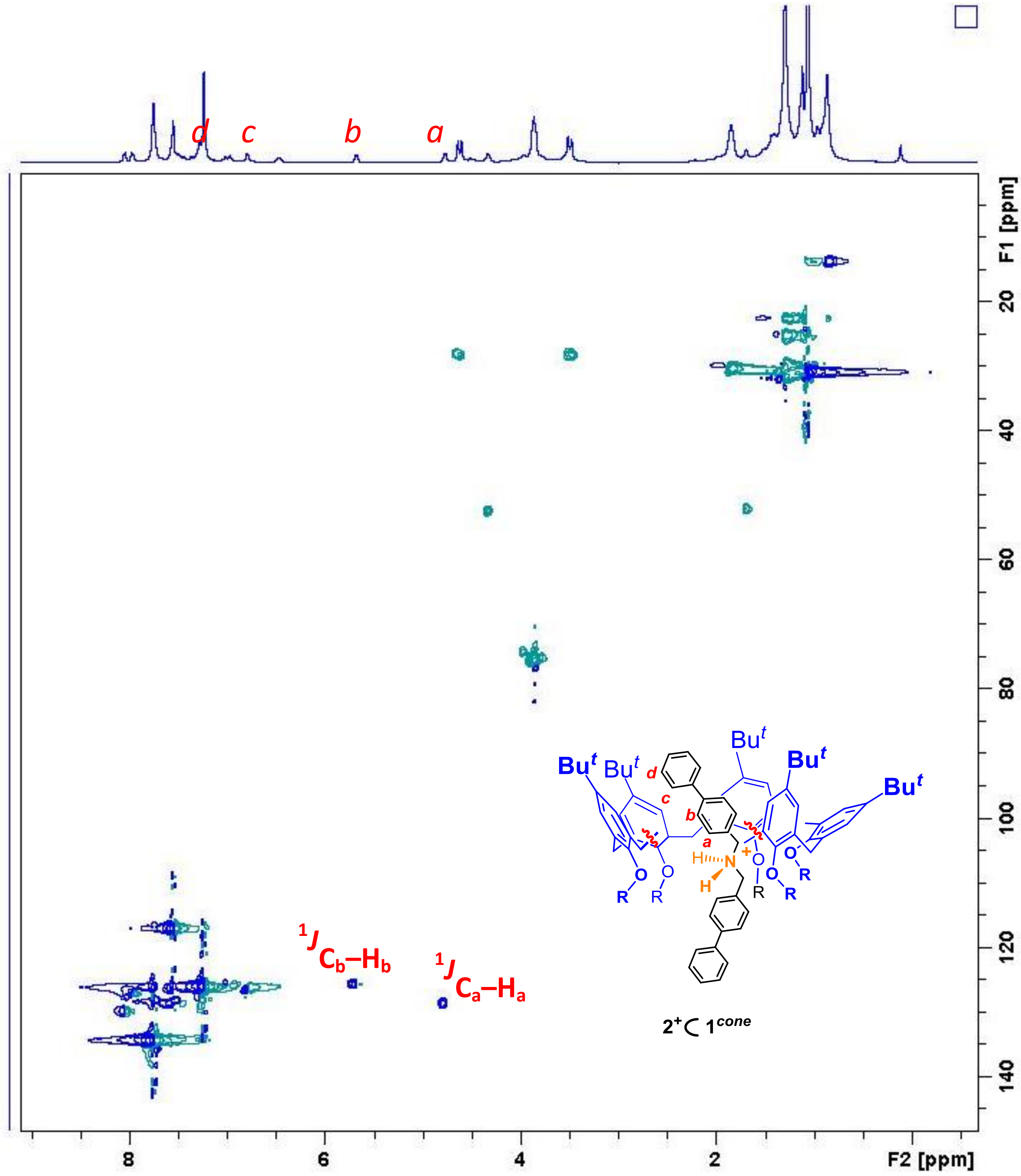

Figure S8: HSQC spectrum (600 MHz, $CDCl_3$, 298 K) of a 1:1 mixture (0.003 M) of **1** and $\mathbf{2^{+}}$**·TFPB⁻** after 18 h by mixing, $\mathbf{2^{+}\subset 1^{cone}}$.

2D COSY spectrum of $3^+ \subset 1^{cone}$ atropoisomer at 298 K ($CDCl_3$, 600 MHz)

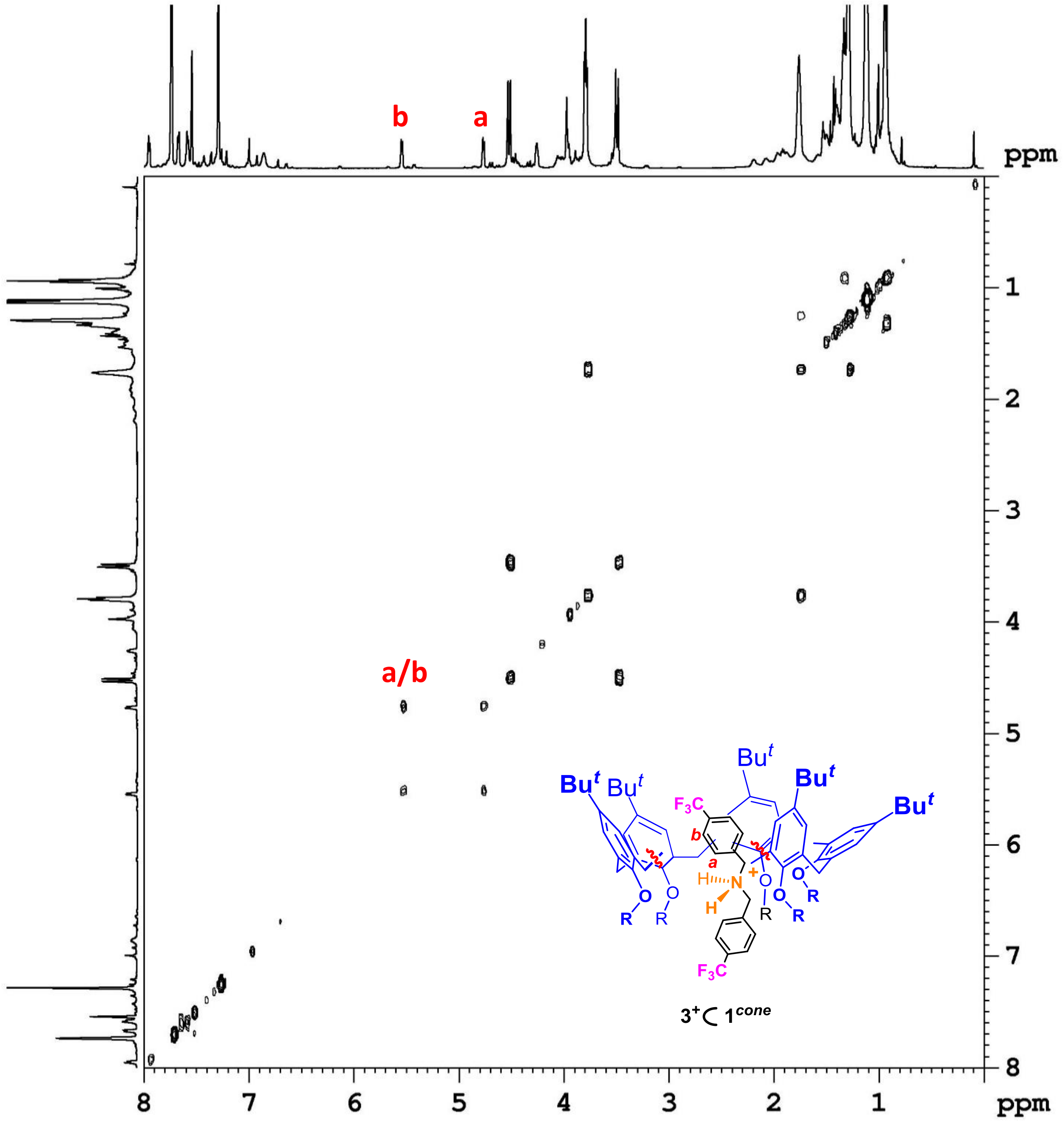

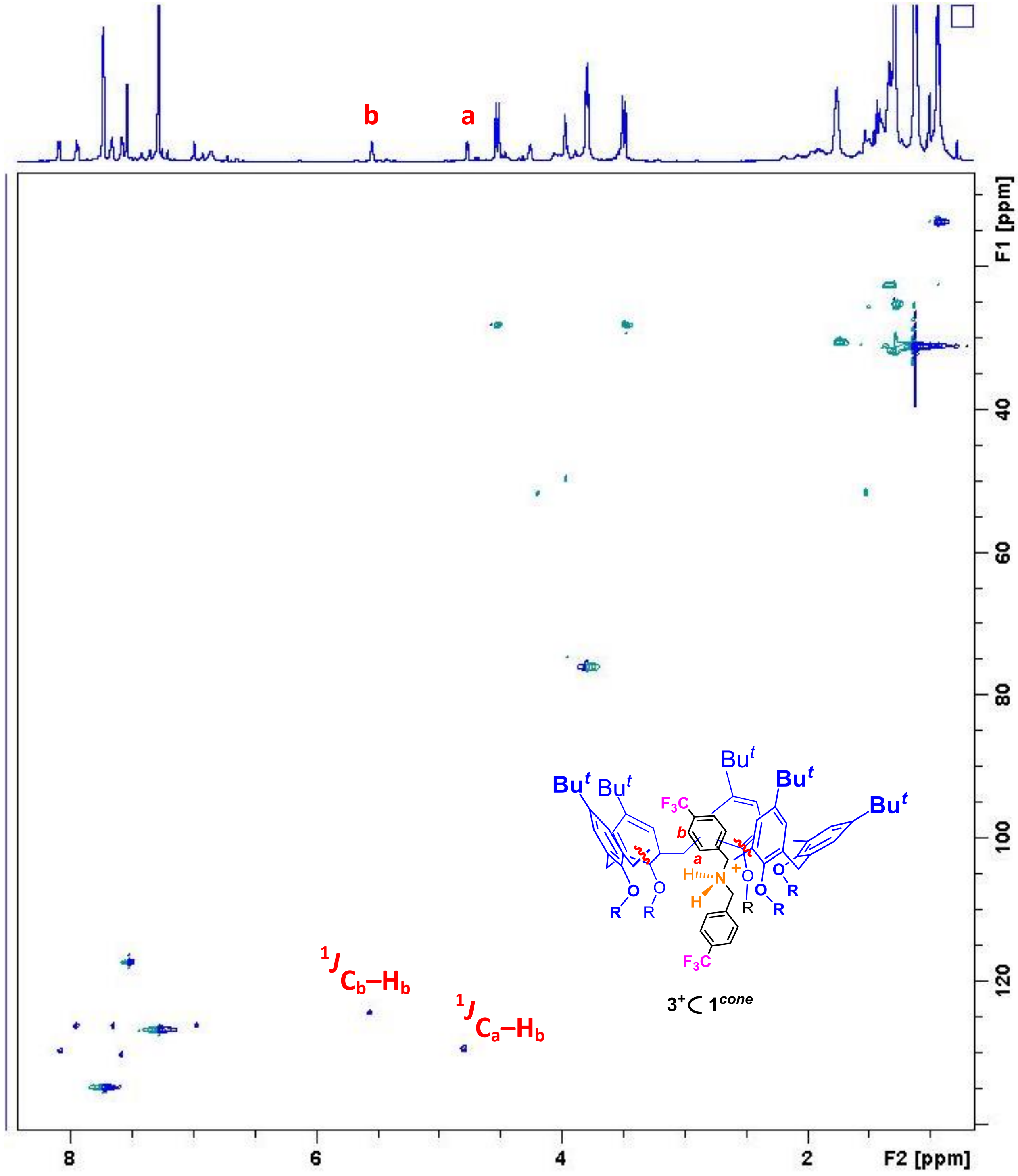

Figure S10: HSQC spectrum (600 MHz, $CDCl_3$, 298 K) of a 1:1 mixture (0.003 M) of **1** and **3^+·TFPB$^-$** after 24 h, **$3^+ \subset 1^{cone}$**.

Determination of apparent association constant values by quantitative ^{1}H NMR analysis (for pseudorotaxanes $\mathbf{2^{+}\subset 1^{\mathit{cone}}}$, $\mathbf{3^{+}\subset 1^{\mathit{cone}}}$ and $\mathbf{3^{+}\subset 1^{\mathit{1,2,3\text{-}alt}}}$).

The samples were prepared by dissolving calixarene-**1** (3.0×10^{-3} M) and the appropriate ammonium TFPB salt (**2^{+}** or **3^{+}**) (3.0×10^{-3} M) in $CDCl_3$ (0.5 mL) containing 0.10 μL of TCHE (d = 1.596 g/mL) as an internal standard. The complex concentration [complex] was evaluated by integration of the ^{1}H NMR signals of TCHE versus the signals of the pseudorotaxane. The following equation was used to obtain the moles of the complex:

$$\frac{G_a}{G_b} = \frac{F_a}{F_b} \times \frac{N_b}{N_a} \times \frac{M_a}{M_b}$$

in which:

G_a = grams of TCHE; G_b= grams of pseudorotaxane

F_a and F_b = areas of the signals of, TCHE and shielded aromatic protons of the axle.

N_a and N_b = are the numbers of nuclei that cause the signals (N_a for TCHE; N_b for pseudorotaxane)

M_a and M_b = are the molecular masses of TCHE (a) and pseudorotaxane (b)

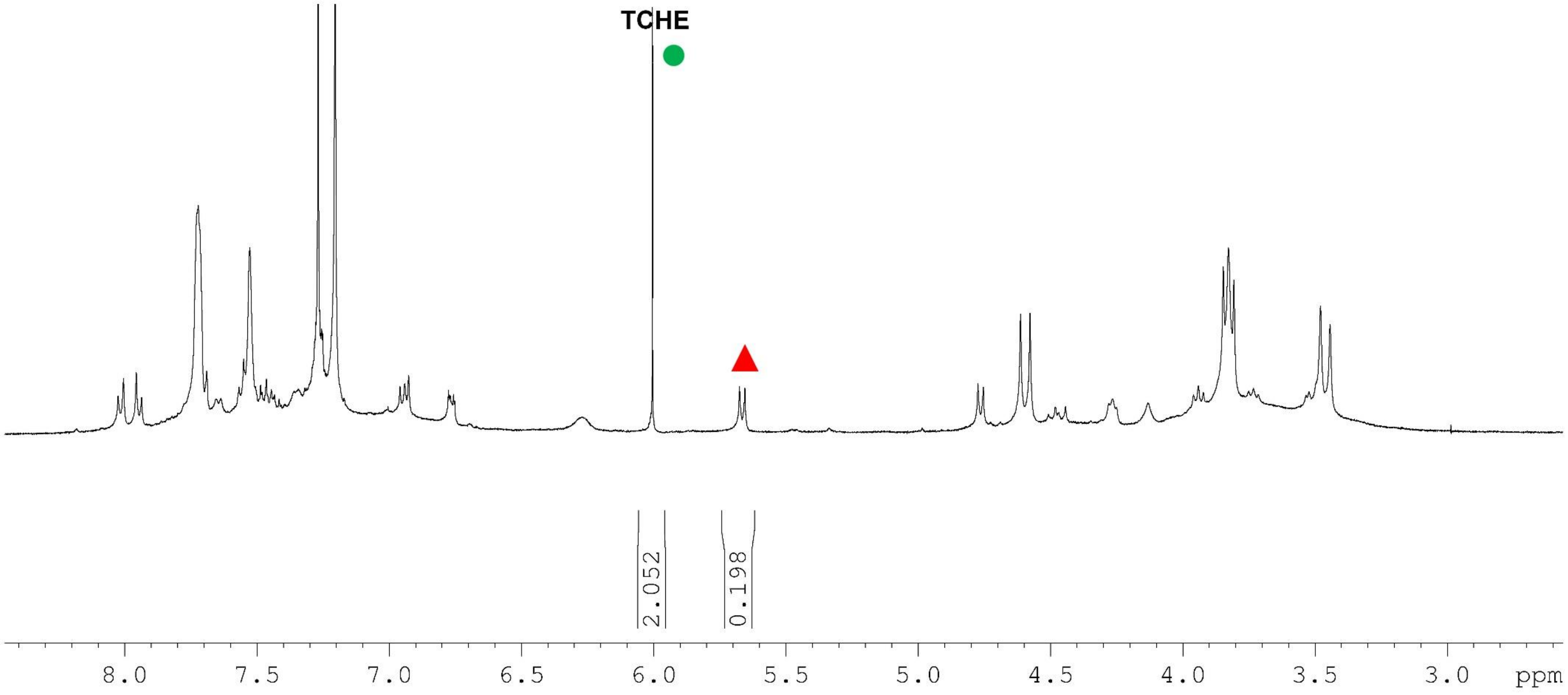

Figure S11. ^{1}H NMR spectra (600 MHz, $CDCl_3$, 298 K) of a 1:1 mixture (0.003 M) of hexahexyloxycalix[6]arene **1** and **2^+·TFPB$^-$** in 0.5 mL of $CDCl_3$ containing 1.0 μL of TCHE after equilibration for 48 h at 298 K. The association constant K_a values were calculated by integration of complexed Ar*H* signal of $\mathbf{2}^+\subset\mathbf{1}^{cone}$ (▲) and signal of TCHE (●).

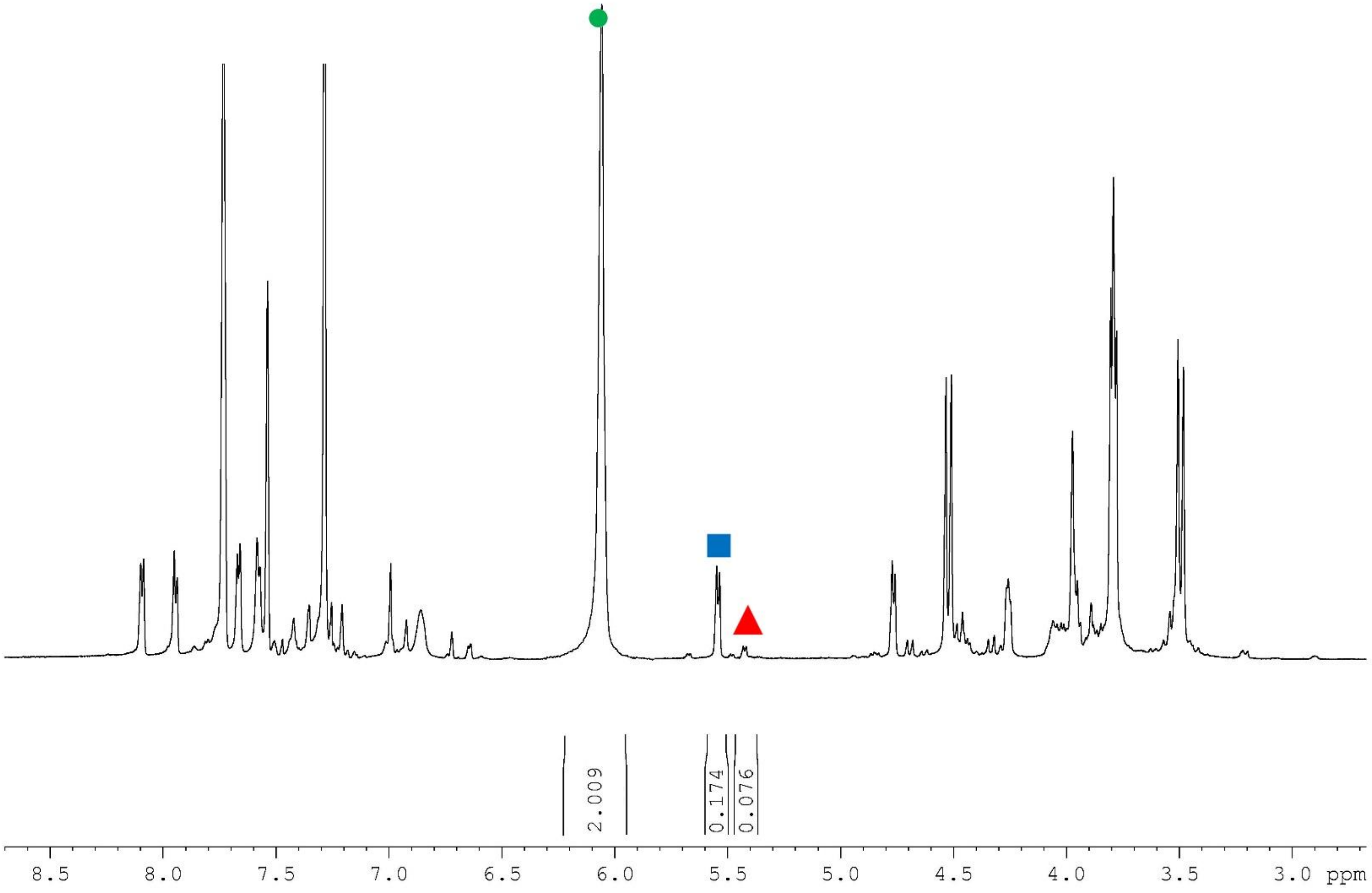

Figure S12. ^{1}H NMR spectra (600 MHz, $CDCl_3$, 298 K) of a 1:1 mixture (0.0024 M) of hexahexyloxycalix[6]arene **1** and **2^{+}·TFPB^{-}** in 0.5 mL of $CDCl_3$ containing 0.10 μL of TCHE after equilibration for 48 h at 298 K. The association constant K_a values were calculated by integration of complexed Ar*H* signals of **$3^{+}\subset1^{cone}$**(■), **$3^{+}\subset1^{1,2,3\text{-}alt}$** (▲) and signal of TCHE (●).

DFT Calculations.

Preliminary conformational searches have been performed by molecular mechanics calculations (Spartan 02). The minimized structures were optimized by DFT calculations (Gaussian 09) at B3LYP/6-31G(d,p) level of theory using Grimme's dispersion corrections

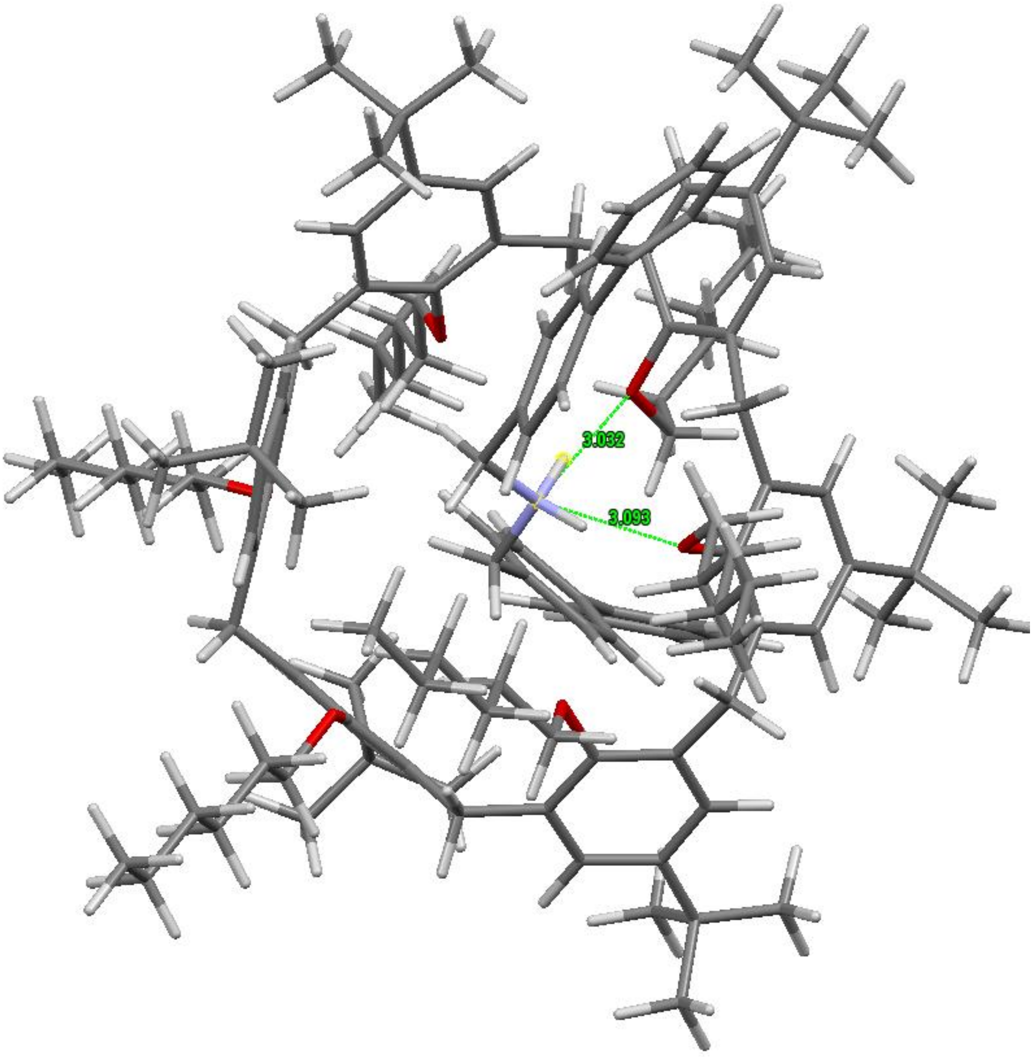

(IOp(3/124 = 3)).

Figure S13: DFT-optimized structure of the $\mathbf{2^{+}}\subset\mathbf{1}^{\mathit{cone}}$ atropoisomer with H-bonds N···O distances indicated.

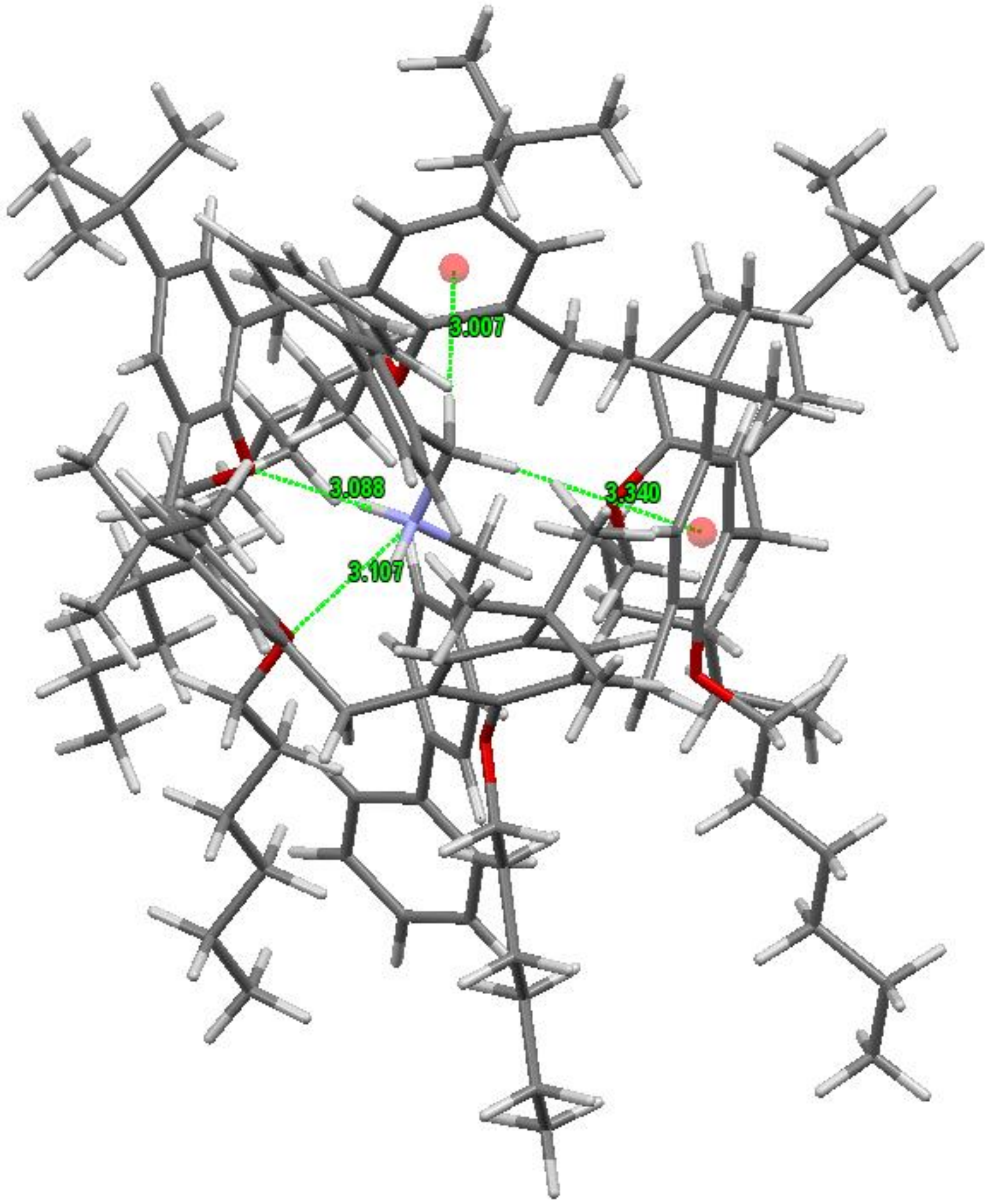

Figure S14: DFT-optimized structure of the $\mathbf{2^{+}{\subset}1^{\mathit{cone}}}$ atropoisomer with C-H⋯π distances indicated.

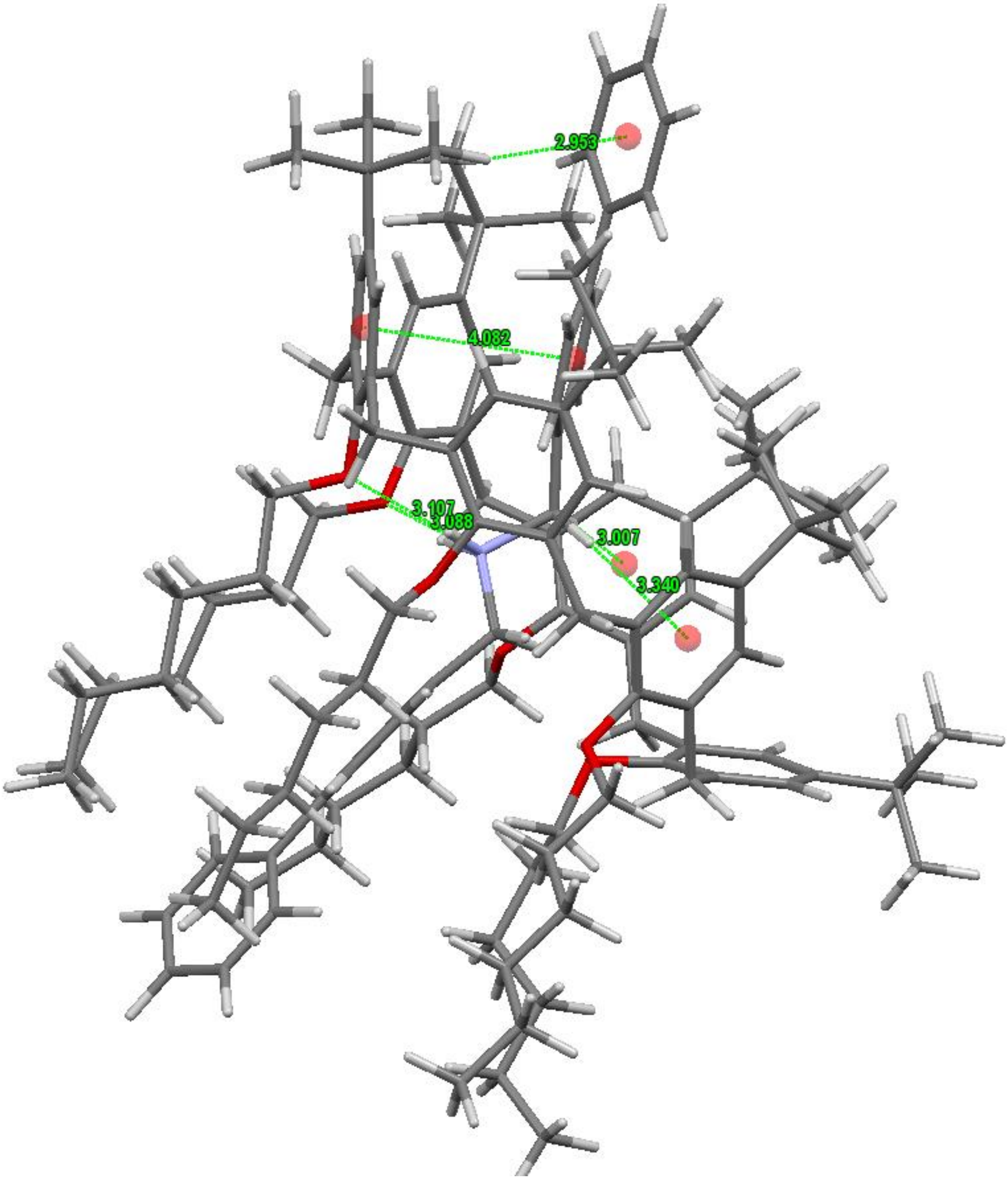

Figure S15: DFT-optimized structure of the $2^+\subset1^{cone}$ atropoisomer with C-H···π and π ···π distances indicated.

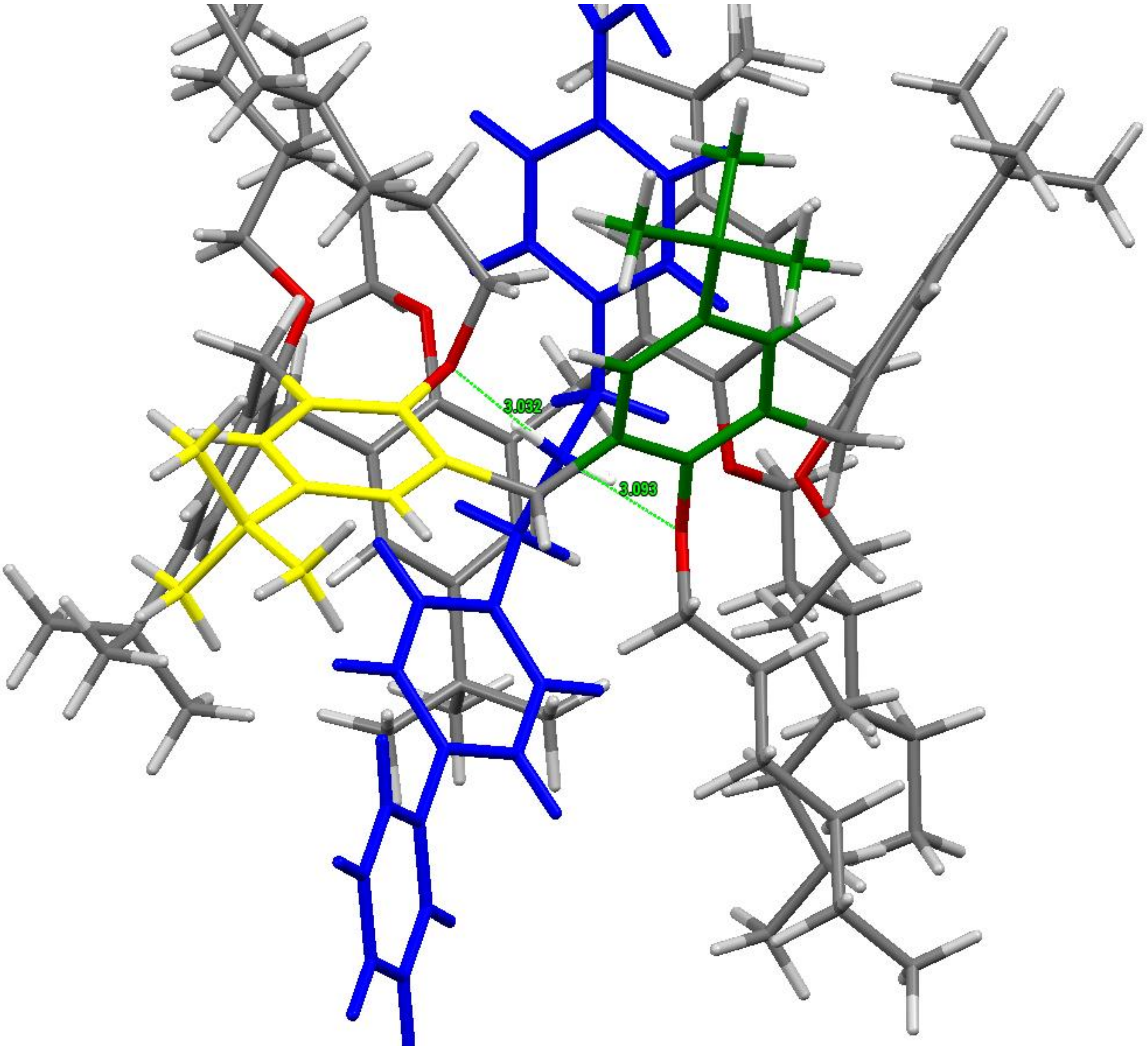

Figure S16: DFT-optimized structure of the $\mathbf{2^{+}\subset 1}^{\mathit{1,2,3\text{-}alt}}$ atropoisomer with H-bonds N···O distances indicated.

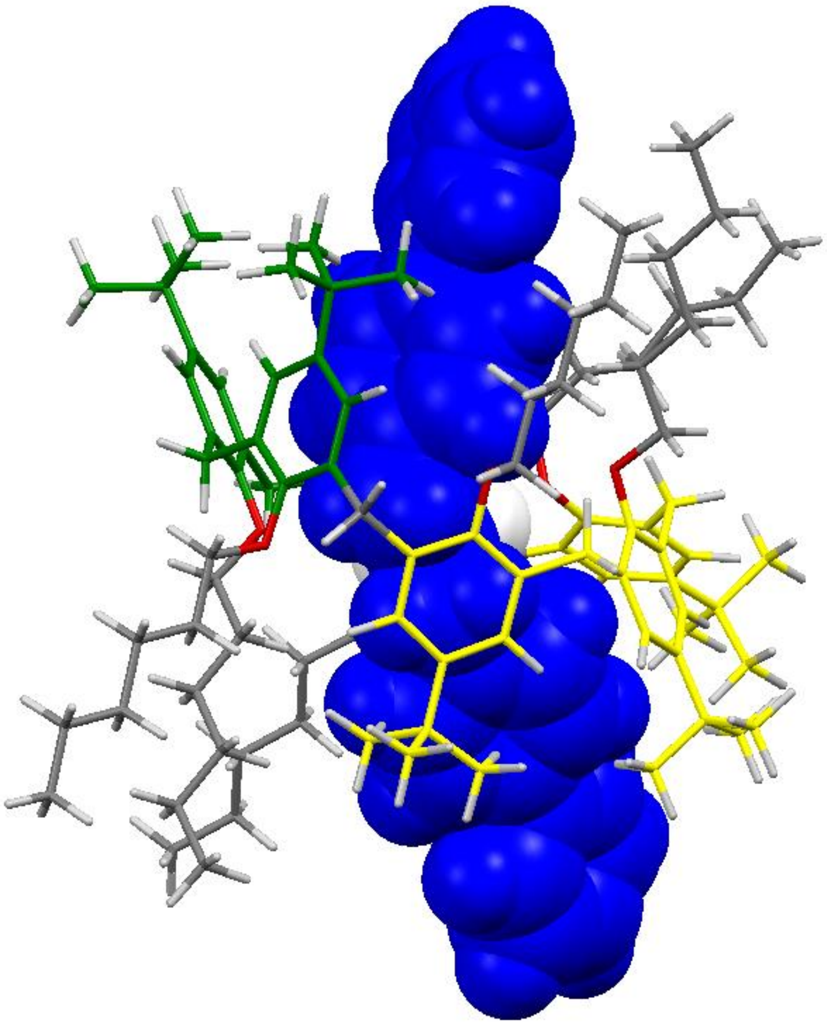

Figure S17: Different view of the DFT-optimized structure of the $\mathbf{2^+{\subset}1^{\mathit{1,2,3\text{-}alt}}}$ atropoisomer.

Atomic coordinates of **2**$^{+}$⊂**1**^{cone}

HETATM	1	O1	UNK	1	0.141	4.032	-0.402	1.00	0.00	O
HETATM	2	O2	UNK	1	-2.211	2.787	2.878	1.00	0.00	O
HETATM	3	O3	UNK	1	-1.376	-4.335	-0.395	1.00	0.00	O
HETATM	4	O4	UNK	1	-2.721	-1.382	2.257	1.00	0.00	O
HETATM	5	O5	UNK	1	0.881	-2.257	-2.796	1.00	0.00	O
HETATM	6	C6	UNK	1	-2.446	-1.914	3.508	1.00	0.00	C
HETATM	7	C7	UNK	1	1.273	1.248	4.666	1.00	0.00	C
HETATM	8	C8	UNK	1	-1.063	2.288	3.479	1.00	0.00	C
HETATM	9	C9	UNK	1	0.034	0.622	4.834	1.00	0.00	C
HETATM	10	C10	UNK	1	1.312	2.393	3.856	1.00	0.00	C
HETATM	11	C11	UNK	1	-2.420	-1.070	4.635	1.00	0.00	C
HETATM	12	C12	UNK	1	-2.014	-4.169	2.406	1.00	0.00	C
HETATM	13	C13	UNK	1	2.196	-2.720	-2.657	1.00	0.00	C
HETATM	14	C14	UNK	1	0.985	-4.628	0.024	1.00	0.00	C
HETATM	15	C15	UNK	1	-2.491	0.441	4.500	1.00	0.00	C
HETATM	16	C16	UNK	1	-0.325	-4.438	0.510	1.00	0.00	C
HETATM	17	C17	UNK	1	-2.250	-1.653	5.893	1.00	0.00	C
HETATM	18	C18	UNK	1	3.738	4.723	0.028	1.00	0.00	C
HETATM	19	C19	UNK	1	-0.581	-4.338	1.890	1.00	0.00	C
HETATM	20	C20	UNK	1	1.435	4.453	1.590	1.00	0.00	C
HETATM	21	C21	UNK	1	3.817	5.028	1.389	1.00	0.00	C
HETATM	22	C22	UNK	1	2.982	-0.658	-3.929	1.00	0.00	C
HETATM	23	C23	UNK	1	-1.141	1.118	4.255	1.00	0.00	C
HETATM	24	C24	UNK	1	0.213	4.246	2.466	1.00	0.00	C
HETATM	25	C25	UNK	1	1.804	-4.714	2.339	1.00	0.00	C
HETATM	26	C26	UNK	1	1.246	-4.781	-1.475	1.00	0.00	C
HETATM	27	C27	UNK	1	0.497	-4.471	2.774	1.00	0.00	C
HETATM	28	C28	UNK	1	0.170	2.936	3.262	1.00	0.00	C
HETATM	29	C29	UNK	1	3.701	-4.488	-2.049	1.00	0.00	C
HETATM	30	C30	UNK	1	2.538	4.342	-0.595	1.00	0.00	C
HETATM	31	C31	UNK	1	4.540	-2.533	-3.146	1.00	0.00	C
HETATM	32	C32	UNK	1	-2.163	-3.282	3.629	1.00	0.00	C
HETATM	33	C33	UNK	1	3.256	-1.969	-3.189	1.00	0.00	C
HETATM	34	C34	UNK	1	4.790	-3.800	-2.608	1.00	0.00	C
HETATM	35	C35	UNK	1	2.402	-3.973	-2.049	1.00	0.00	C
HETATM	36	C36	UNK	1	1.380	4.257	0.194	1.00	0.00	C
HETATM	37	C37	UNK	1	2.021	-4.765	0.954	1.00	0.00	C
HETATM	38	C38	UNK	1	-2.062	-3.033	6.072	1.00	0.00	C
HETATM	39	C39	UNK	1	-1.996	-3.817	4.916	1.00	0.00	C
HETATM	40	C40	UNK	1	2.648	4.864	2.149	1.00	0.00	C
HETATM	41	C41	UNK	1	2.491	4.090	-2.102	1.00	0.00	C
HETATM	42	C42	UNK	1	4.726	3.143	-2.846	1.00	0.00	C
HETATM	43	C43	UNK	1	5.563	2.141	-3.361	1.00	0.00	C
HETATM	44	C44	UNK	1	4.980	0.900	-3.633	1.00	0.00	C
HETATM	45	C45	UNK	1	3.613	0.648	-3.447	1.00	0.00	C
HETATM	46	C46	UNK	1	2.814	1.693	-2.954	1.00	0.00	C
HETATM	47	C47	UNK	1	3.361	2.947	-2.620	1.00	0.00	C
HETATM	48	O48	UNK	1	1.427	1.522	-2.864	1.00	0.00	O
HETATM	49	C49	UNK	1	0.322	-2.701	-4.064	1.00	0.00	C
HETATM	50	H50	UNK	1	-0.041	-0.277	5.432	1.00	0.00	H
HETATM	51	H51	UNK	1	2.261	2.888	3.689	1.00	0.00	H
HETATM	52	H52	UNK	1	-2.621	-3.781	1.590	1.00	0.00	H
HETATM	53	H53	UNK	1	-2.407	-5.165	2.651	1.00	0.00	H
HETATM	54	H54	UNK	1	-2.921	0.848	5.425	1.00	0.00	H
HETATM	55	H55	UNK	1	-3.160	0.741	3.693	1.00	0.00	H

HETATM	56	H56	UNK	1	-4.104	-0.287	1.249	1.00	0.00	H
HETATM	57	H57	UNK	1	4.622	4.811	-0.590	1.00	0.00	H
HETATM	58	H58	UNK	1	0.157	5.077	3.183	1.00	0.00	H
HETATM	59	H59	UNK	1	-0.690	4.292	1.859	1.00	0.00	H
HETATM	60	H60	UNK	1	-2.474	-5.982	0.262	1.00	0.00	H
HETATM	61	H61	UNK	1	0.290	-4.396	3.834	1.00	0.00	H
HETATM	62	H62	UNK	1	3.852	-5.482	-1.638	1.00	0.00	H
HETATM	63	H63	UNK	1	3.023	-4.943	0.578	1.00	0.00	H
HETATM	64	H64	UNK	1	-1.829	-4.885	4.998	1.00	0.00	H
HETATM	65	H65	UNK	1	2.805	5.008	-2.613	1.00	0.00	H
HETATM	66	H66	UNK	1	1.454	3.906	-2.375	1.00	0.00	H
HETATM	67	H67	UNK	1	1.905	-0.516	-3.940	1.00	0.00	H
HETATM	68	H68	UNK	1	3.297	-0.803	-4.971	1.00	0.00	H
HETATM	69	H69	UNK	1	0.324	-4.527	-1.997	1.00	0.00	H
HETATM	70	H70	UNK	1	1.447	-5.840	-1.678	1.00	0.00	H
HETATM	71	H71	UNK	1	5.589	0.113	-4.061	1.00	0.00	H
HETATM	72	H72	UNK	1	5.354	-1.974	-3.587	1.00	0.00	H
HETATM	73	H73	UNK	1	2.667	5.087	3.212	1.00	0.00	H
HETATM	74	H74	UNK	1	-2.264	-0.997	6.760	1.00	0.00	H
HETATM	75	H75	UNK	1	5.138	4.129	-2.657	1.00	0.00	H
HETATM	76	H76	UNK	1	0.437	-3.790	-4.126	1.00	0.00	H
HETATM	77	H77	UNK	1	0.913	-2.264	-4.878	1.00	0.00	H
HETATM	78	C78	UNK	1	-2.966	3.717	3.676	1.00	0.00	C
HETATM	79	C79	UNK	1	-4.116	-1.059	2.023	1.00	0.00	C
HETATM	80	C80	UNK	1	-0.509	5.276	-0.756	1.00	0.00	C
HETATM	81	C81	UNK	1	0.774	1.924	-4.097	1.00	0.00	C
HETATM	82	C82	UNK	1	-2.050	-5.581	-0.668	1.00	0.00	C
HETATM	83	H83	UNK	1	0.009	5.721	-1.617	1.00	0.00	H
HETATM	84	H84	UNK	1	-3.227	3.256	4.638	1.00	0.00	H
HETATM	85	H85	UNK	1	-4.541	-0.626	2.933	1.00	0.00	H
HETATM	86	H86	UNK	1	-0.416	5.980	0.081	1.00	0.00	H
HETATM	87	H87	UNK	1	1.186	2.891	-4.410	1.00	0.00	H
HETATM	88	H88	UNK	1	1.020	1.198	-4.882	1.00	0.00	H
HETATM	89	H89	UNK	1	-1.324	-6.314	-1.044	1.00	0.00	H
HETATM	90	H90	UNK	1	-2.353	4.604	3.890	1.00	0.00	H
HETATM	91	C109	UNK	1	6.169	-4.484	-2.682	1.00	0.00	C
HETATM	92	C110	UNK	1	6.646	-4.887	-1.268	1.00	0.00	C
HETATM	93	H111	UNK	1	6.779	-4.009	-0.628	1.00	0.00	H
HETATM	94	H112	UNK	1	5.941	-5.560	-0.771	1.00	0.00	H
HETATM	95	H113	UNK	1	7.609	-5.406	-1.325	1.00	0.00	H
HETATM	96	C114	UNK	1	6.048	-5.758	-3.555	1.00	0.00	C
HETATM	97	H115	UNK	1	5.339	-6.476	-3.132	1.00	0.00	H
HETATM	98	H116	UNK	1	5.711	-5.509	-4.566	1.00	0.00	H
HETATM	99	H117	UNK	1	7.020	-6.258	-3.634	1.00	0.00	H
HETATM	100	C118	UNK	1	7.241	-3.576	-3.314	1.00	0.00	C
HETATM	101	H119	UNK	1	8.203	-4.098	-3.330	1.00	0.00	H
HETATM	102	H120	UNK	1	6.995	-3.312	-4.347	1.00	0.00	H
HETATM	103	H121	UNK	1	7.378	-2.649	-2.748	1.00	0.00	H
HETATM	104	C122	UNK	1	-1.934	-3.616	7.492	1.00	0.00	C
HETATM	105	C123	UNK	1	-1.730	-5.143	7.481	1.00	0.00	C
HETATM	106	H124	UNK	1	-2.570	-5.664	7.010	1.00	0.00	H
HETATM	107	H125	UNK	1	-0.811	-5.430	6.959	1.00	0.00	H
HETATM	108	H126	UNK	1	-1.651	-5.511	8.508	1.00	0.00	H
HETATM	109	C127	UNK	1	-0.725	-2.974	8.212	1.00	0.00	C
HETATM	110	H128	UNK	1	0.207	-3.180	7.674	1.00	0.00	H
HETATM	111	H129	UNK	1	-0.829	-1.889	8.301	1.00	0.00	H
HETATM	112	H130	UNK	1	-0.626	-3.380	9.225	1.00	0.00	H
HETATM	113	C131	UNK	1	-3.225	-3.307	8.287	1.00	0.00	C
HETATM	114	H132	UNK	1	-3.151	-3.708	9.304	1.00	0.00	H
HETATM	115	H133	UNK	1	-3.407	-2.231	8.366	1.00	0.00	H
HETATM	116	H134	UNK	1	-4.099	-3.760	7.807	1.00	0.00	H
HETATM	117	C135	UNK	1	2.969	-4.982	3.313	1.00	0.00	C

HETATM	118	C136	UNK	1	2.548	-4.822	4.786	1.00	0.00	C
HETATM	119	H137	UNK	1	2.189	-3.810	5.001	1.00	0.00	H
HETATM	120	H138	UNK	1	1.763	-5.531	5.067	1.00	0.00	H
HETATM	121	H139	UNK	1	3.407	-5.012	5.436	1.00	0.00	H
HETATM	122	C140	UNK	1	4.138	-4.010	3.039	1.00	0.00	C
HETATM	123	H141	UNK	1	3.830	-2.968	3.170	1.00	0.00	H
HETATM	124	H142	UNK	1	4.963	-4.207	3.733	1.00	0.00	H
HETATM	125	H143	UNK	1	4.533	-4.117	2.025	1.00	0.00	H
HETATM	126	C144	UNK	1	3.461	-6.436	3.109	1.00	0.00	C
HETATM	127	H145	UNK	1	4.288	-6.658	3.791	1.00	0.00	H
HETATM	128	H146	UNK	1	2.657	-7.153	3.304	1.00	0.00	H
HETATM	129	H147	UNK	1	3.817	-6.603	2.087	1.00	0.00	H
HETATM	130	C148	UNK	1	2.556	0.751	5.361	1.00	0.00	C
HETATM	131	C149	UNK	1	2.310	-0.511	6.209	1.00	0.00	C
HETATM	132	H150	UNK	1	1.942	-1.344	5.601	1.00	0.00	H
HETATM	133	H151	UNK	1	3.247	-0.829	6.675	1.00	0.00	H
HETATM	134	H152	UNK	1	1.588	-0.330	7.012	1.00	0.00	H
HETATM	135	C153	UNK	1	3.085	1.862	6.299	1.00	0.00	C
HETATM	136	H154	UNK	1	2.342	2.119	7.061	1.00	0.00	H
HETATM	137	H155	UNK	1	3.995	1.529	6.810	1.00	0.00	H
HETATM	138	H156	UNK	1	3.329	2.776	5.749	1.00	0.00	H
HETATM	139	C157	UNK	1	3.637	0.419	4.308	1.00	0.00	C
HETATM	140	H158	UNK	1	4.560	0.094	4.800	1.00	0.00	H
HETATM	141	H159	UNK	1	3.308	-0.388	3.644	1.00	0.00	H
HETATM	142	H160	UNK	1	3.881	1.281	3.681	1.00	0.00	H
HETATM	143	C161	UNK	1	5.099	5.557	2.060	1.00	0.00	C
HETATM	144	C162	UNK	1	6.290	5.621	1.085	1.00	0.00	C
HETATM	145	H163	UNK	1	6.093	6.293	0.243	1.00	0.00	H
HETATM	146	H164	UNK	1	7.172	6.005	1.607	1.00	0.00	H
HETATM	147	H165	UNK	1	6.551	4.636	0.683	1.00	0.00	H
HETATM	148	C166	UNK	1	4.836	6.986	2.591	1.00	0.00	C
HETATM	149	H167	UNK	1	4.570	7.665	1.775	1.00	0.00	H
HETATM	150	H168	UNK	1	4.020	7.003	3.321	1.00	0.00	H
HETATM	151	H169	UNK	1	5.731	7.381	3.083	1.00	0.00	H
HETATM	152	C170	UNK	1	5.493	4.642	3.243	1.00	0.00	C
HETATM	153	H171	UNK	1	6.415	5.002	3.712	1.00	0.00	H
HETATM	154	H172	UNK	1	4.720	4.617	4.017	1.00	0.00	H
HETATM	155	H173	UNK	1	5.662	3.613	2.908	1.00	0.00	H
HETATM	156	C174	UNK	1	7.035	2.464	-3.689	1.00	0.00	C
HETATM	157	C175	UNK	1	7.069	3.492	-4.845	1.00	0.00	C
HETATM	158	H176	UNK	1	6.577	3.096	-5.740	1.00	0.00	H
HETATM	159	H177	UNK	1	6.565	4.425	-4.573	1.00	0.00	H
HETATM	160	H178	UNK	1	8.104	3.736	-5.107	1.00	0.00	H
HETATM	161	C179	UNK	1	7.744	3.064	-2.454	1.00	0.00	C
HETATM	162	H180	UNK	1	7.268	3.990	-2.116	1.00	0.00	H
HETATM	163	H181	UNK	1	7.749	2.359	-1.618	1.00	0.00	H
HETATM	164	H182	UNK	1	8.784	3.304	-2.699	1.00	0.00	H
HETATM	165	C183	UNK	1	7.823	1.215	-4.130	1.00	0.00	C
HETATM	166	H184	UNK	1	8.863	1.490	-4.327	1.00	0.00	H
HETATM	167	H185	UNK	1	7.827	0.445	-3.352	1.00	0.00	H
HETATM	168	H186	UNK	1	7.423	0.779	-5.051	1.00	0.00	H
HETATM	169	C187	UNK	1	-1.138	-2.314	-4.198	1.00	0.00	C
HETATM	170	H188	UNK	1	-1.708	-2.768	-3.381	1.00	0.00	H
HETATM	171	H189	UNK	1	-1.245	-1.228	-4.094	1.00	0.00	H
HETATM	172	C190	UNK	1	-1.699	-2.770	-5.554	1.00	0.00	C
HETATM	173	H191	UNK	1	-1.114	-2.311	-6.363	1.00	0.00	H
HETATM	174	H192	UNK	1	-1.560	-3.855	-5.659	1.00	0.00	H
HETATM	175	C193	UNK	1	-3.181	-2.433	-5.753	1.00	0.00	C
HETATM	176	H194	UNK	1	-3.770	-2.904	-4.953	1.00	0.00	H
HETATM	177	H195	UNK	1	-3.325	-1.348	-5.643	1.00	0.00	H
HETATM	178	C196	UNK	1	-3.729	-2.880	-7.114	1.00	0.00	C
HETATM	179	H197	UNK	1	-3.140	-2.410	-7.913	1.00	0.00	H

HETATM	180	H198	UNK	1	-3.581	-3.962	-7.225	1.00	0.00	H
HETATM	181	C199	UNK	1	-5.211	-2.545	-7.308	1.00	0.00	C
HETATM	182	H200	UNK	1	-5.831	-3.034	-6.548	1.00	0.00	H
HETATM	183	H201	UNK	1	-5.385	-1.465	-7.238	1.00	0.00	H
HETATM	184	H202	UNK	1	-5.569	-2.874	-8.288	1.00	0.00	H
HETATM	185	C203	UNK	1	-0.728	2.031	-3.912	1.00	0.00	C
HETATM	186	H204	UNK	1	-1.133	1.068	-3.580	1.00	0.00	H
HETATM	187	H205	UNK	1	-0.937	2.748	-3.111	1.00	0.00	H
HETATM	188	C206	UNK	1	-1.408	2.470	-5.219	1.00	0.00	C
HETATM	189	H207	UNK	1	-0.947	3.403	-5.572	1.00	0.00	H
HETATM	190	H208	UNK	1	-1.209	1.723	-6.000	1.00	0.00	H
HETATM	191	C209	UNK	1	-2.922	2.678	-5.097	1.00	0.00	C
HETATM	192	H210	UNK	1	-3.393	1.756	-4.732	1.00	0.00	H
HETATM	193	H211	UNK	1	-3.122	3.441	-4.332	1.00	0.00	H
HETATM	194	C212	UNK	1	-3.583	3.101	-6.415	1.00	0.00	C
HETATM	195	H213	UNK	1	-3.099	4.016	-6.785	1.00	0.00	H
HETATM	196	H214	UNK	1	-3.395	2.332	-7.176	1.00	0.00	H
HETATM	197	C215	UNK	1	-5.091	3.336	-6.287	1.00	0.00	C
HETATM	198	H216	UNK	1	-5.608	2.432	-5.948	1.00	0.00	H
HETATM	199	H217	UNK	1	-5.307	4.129	-5.562	1.00	0.00	H
HETATM	200	H218	UNK	1	-5.529	3.635	-7.244	1.00	0.00	H
HETATM	201	C219	UNK	1	-4.946	-2.257	1.569	1.00	0.00	C
HETATM	202	H220	UNK	1	-4.467	-2.693	0.684	1.00	0.00	H
HETATM	203	H221	UNK	1	-4.938	-3.025	2.352	1.00	0.00	H
HETATM	204	C222	UNK	1	-6.400	-1.889	1.221	1.00	0.00	C
HETATM	205	H223	UNK	1	-6.402	-1.110	0.446	1.00	0.00	H
HETATM	206	H224	UNK	1	-6.874	-2.766	0.764	1.00	0.00	H
HETATM	207	C225	UNK	1	-7.259	-1.431	2.409	1.00	0.00	C
HETATM	208	H226	UNK	1	-7.214	-2.190	3.203	1.00	0.00	H
HETATM	209	H227	UNK	1	-6.846	-0.511	2.846	1.00	0.00	H
HETATM	210	C228	UNK	1	-8.725	-1.183	2.030	1.00	0.00	C
HETATM	211	H229	UNK	1	-8.768	-0.434	1.227	1.00	0.00	H
HETATM	212	H230	UNK	1	-9.147	-2.104	1.605	1.00	0.00	H
HETATM	213	C231	UNK	1	-9.585	-0.721	3.209	1.00	0.00	C
HETATM	214	H232	UNK	1	-9.589	-1.465	4.013	1.00	0.00	H
HETATM	215	H233	UNK	1	-9.210	0.218	3.631	1.00	0.00	H
HETATM	216	H234	UNK	1	-10.624	-0.556	2.905	1.00	0.00	H
HETATM	217	C235	UNK	1	-3.145	-5.329	-1.693	1.00	0.00	C
HETATM	218	H236	UNK	1	-3.821	-4.557	-1.303	1.00	0.00	H
HETATM	219	H237	UNK	1	-2.689	-4.920	-2.604	1.00	0.00	H
HETATM	220	C238	UNK	1	-3.943	-6.594	-2.035	1.00	0.00	C
HETATM	221	H239	UNK	1	-3.259	-7.373	-2.397	1.00	0.00	H
HETATM	222	H240	UNK	1	-4.404	-6.994	-1.121	1.00	0.00	H
HETATM	223	C241	UNK	1	-5.033	-6.349	-3.086	1.00	0.00	C
HETATM	224	H242	UNK	1	-5.714	-5.564	-2.727	1.00	0.00	H
HETATM	225	H243	UNK	1	-4.570	-5.954	-4.002	1.00	0.00	H
HETATM	226	C244	UNK	1	-5.845	-7.603	-3.431	1.00	0.00	C
HETATM	227	H245	UNK	1	-5.165	-8.389	-3.785	1.00	0.00	H
HETATM	228	H246	UNK	1	-6.311	-7.994	-2.517	1.00	0.00	H
HETATM	229	C247	UNK	1	-6.925	-7.348	-4.487	1.00	0.00	C
HETATM	230	H248	UNK	1	-7.642	-6.592	-4.147	1.00	0.00	H
HETATM	231	H249	UNK	1	-6.487	-6.990	-5.425	1.00	0.00	H
HETATM	232	H250	UNK	1	-7.487	-8.260	-4.710	1.00	0.00	H
HETATM	233	C251	UNK	1	-1.975	5.031	-1.075	1.00	0.00	C
HETATM	234	H252	UNK	1	-2.055	4.312	-1.899	1.00	0.00	H
HETATM	235	H253	UNK	1	-2.454	4.570	-0.202	1.00	0.00	H
HETATM	236	C254	UNK	1	-2.709	6.328	-1.446	1.00	0.00	C
HETATM	237	H255	UNK	1	-2.608	7.052	-0.625	1.00	0.00	H
HETATM	238	H256	UNK	1	-2.224	6.788	-2.318	1.00	0.00	H
HETATM	239	C257	UNK	1	-4.197	6.114	-1.748	1.00	0.00	C
HETATM	240	H258	UNK	1	-4.302	5.395	-2.574	1.00	0.00	H
HETATM	241	H259	UNK	1	-4.681	5.650	-0.876	1.00	0.00	H

```
HETATM  242 C260 UNK     1      -4.941   7.407  -2.109  1.00  0.00           C
HETATM  243 H261 UNK     1      -4.831   8.127  -1.287  1.00  0.00           H
HETATM  244 H262 UNK     1      -4.460   7.868  -2.982  1.00  0.00           H
HETATM  245 C263 UNK     1      -6.428   7.186  -2.400  1.00  0.00           C
HETATM  246 H264 UNK     1      -6.570   6.495  -3.239  1.00  0.00           H
HETATM  247 H265 UNK     1      -6.944   6.762  -1.531  1.00  0.00           H
HETATM  248 H266 UNK     1      -6.927   8.125  -2.656  1.00  0.00           H
HETATM  249 C267 UNK     1      -4.220   4.099   2.904  1.00  0.00           C
HETATM  250 H268 UNK     1      -3.921   4.522   1.936  1.00  0.00           H
HETATM  251 H269 UNK     1      -4.788   3.187   2.684  1.00  0.00           H
HETATM  252 C270 UNK     1      -5.104   5.100   3.659  1.00  0.00           C
HETATM  253 H271 UNK     1      -5.388   4.678   4.633  1.00  0.00           H
HETATM  254 H272 UNK     1      -4.526   6.009   3.879  1.00  0.00           H
HETATM  255 C273 UNK     1      -6.372   5.483   2.885  1.00  0.00           C
HETATM  256 H274 UNK     1      -6.088   5.899   1.907  1.00  0.00           H
HETATM  257 H275 UNK     1      -6.953   4.575   2.671  1.00  0.00           H
HETATM  258 C276 UNK     1      -7.261   6.491   3.623  1.00  0.00           C
HETATM  259 H277 UNK     1      -7.542   6.077   4.601  1.00  0.00           H
HETATM  260 H278 UNK     1      -6.681   7.399   3.833  1.00  0.00           H
HETATM  261 C279 UNK     1      -8.526   6.861   2.843  1.00  0.00           C
HETATM  262 H280 UNK     1      -8.278   7.309   1.873  1.00  0.00           H
HETATM  263 H281 UNK     1      -9.146   5.978   2.650  1.00  0.00           H
HETATM  264 H282 UNK     1      -9.138   7.582   3.393  1.00  0.00           H
HETATM  265  N91 UNK     1       0.570  -0.255  -0.465  1.00  0.00           N
HETATM  266  C92 UNK     1      -0.798  -0.134   0.202  1.00  0.00           C
HETATM  267  C93 UNK     1      -4.438   0.432  -2.063  1.00  0.00           C
HETATM  268  C94 UNK     1      -3.857  -0.843  -1.937  1.00  0.00           C
HETATM  269  C95 UNK     1      -2.655  -1.025  -1.258  1.00  0.00           C
HETATM  270  C96 UNK     1      -1.986   0.070  -0.694  1.00  0.00           C
HETATM  271  C97 UNK     1      -2.547   1.345  -0.827  1.00  0.00           C
HETATM  272  C98 UNK     1      -3.751   1.524  -1.503  1.00  0.00           C
HETATM  273  H99 UNK     1      -0.923  -1.048   0.784  1.00  0.00           H
HETATM  274 H100 UNK     1      -0.702   0.697   0.903  1.00  0.00           H
HETATM  275 H101 UNK     1       0.824   0.615  -0.957  1.00  0.00           H
HETATM  276 H102 UNK     1       0.557  -0.977  -1.207  1.00  0.00           H
HETATM  277 H103 UNK     1      -4.381  -1.708  -2.331  1.00  0.00           H
HETATM  278 H104 UNK     1      -2.262  -2.029  -1.117  1.00  0.00           H
HETATM  279 H105 UNK     1      -2.046   2.197  -0.378  1.00  0.00           H
HETATM  280 H106 UNK     1      -4.164   2.522  -1.601  1.00  0.00           H
HETATM  281 H107 UNK     1       1.401  -1.686   0.826  1.00  0.00           H
HETATM  282 H108 UNK     1       1.238  -0.066   1.510  1.00  0.00           H
HETATM  283 C283 UNK     1      -8.239   0.960  -4.013  1.00  0.00           C
HETATM  284 C284 UNK     1      -7.907   1.729  -2.896  1.00  0.00           C
HETATM  285 C285 UNK     1      -6.676   1.559  -2.267  1.00  0.00           C
HETATM  286 C286 UNK     1      -5.748   0.615  -2.739  1.00  0.00           C
HETATM  287 C287 UNK     1      -6.097  -0.155  -3.862  1.00  0.00           C
HETATM  288 C288 UNK     1      -7.328   0.016  -4.492  1.00  0.00           C
HETATM  289 H289 UNK     1      -9.198   1.092  -4.503  1.00  0.00           H
HETATM  290 H290 UNK     1      -8.613   2.458  -2.509  1.00  0.00           H
HETATM  291 H291 UNK     1      -6.441   2.146  -1.384  1.00  0.00           H
HETATM  292 H292 UNK     1      -5.390  -0.877  -4.259  1.00  0.00           H
HETATM  293 H293 UNK     1      -7.574  -0.585  -5.363  1.00  0.00           H
HETATM  294 C294 UNK     1       1.574  -0.626   0.634  1.00  0.00           C
HETATM  295 C295 UNK     1       5.827   0.117   0.405  1.00  0.00           C
HETATM  296 C296 UNK     1       4.913   1.183   0.474  1.00  0.00           C
HETATM  297 C297 UNK     1       3.541   0.955   0.487  1.00  0.00           C
HETATM  298 C298 UNK     1       3.033  -0.348   0.396  1.00  0.00           C
HETATM  299 C299 UNK     1       3.935  -1.411   0.286  1.00  0.00           C
HETATM  300 C300 UNK     1       5.308  -1.182   0.299  1.00  0.00           C
HETATM  301 H301 UNK     1       5.274   2.203   0.522  1.00  0.00           H
HETATM  302 H302 UNK     1       2.868   1.801   0.588  1.00  0.00           H
HETATM  303 H303 UNK     1       3.564  -2.428   0.230  1.00  0.00           H
```

```
HETATM  304 H304 UNK     1       5.984  -2.029   0.271  1.00  0.00           H
HETATM  305 C305 UNK     1      10.076   0.787   0.597  1.00  0.00           C
HETATM  306 C306 UNK     1       9.561  -0.235  -0.201  1.00  0.00           C
HETATM  307 C307 UNK     1       8.186  -0.451  -0.264  1.00  0.00           C
HETATM  308 C308 UNK     1       7.292   0.351   0.467  1.00  0.00           C
HETATM  309 C309 UNK     1       7.827   1.373   1.270  1.00  0.00           C
HETATM  310 C310 UNK     1       9.203   1.589   1.333  1.00  0.00           C
HETATM  311 H311 UNK     1      11.148   0.955   0.648  1.00  0.00           H
HETATM  312 H312 UNK     1      10.231  -0.863  -0.781  1.00  0.00           H
HETATM  313 H313 UNK     1       7.797  -1.237  -0.903  1.00  0.00           H
HETATM  314 H314 UNK     1       7.163   1.983   1.873  1.00  0.00           H
HETATM  315 H315 UNK     1       9.594   2.379   1.968  1.00  0.00           H
CONECT    1   36   80
CONECT    2    8   78
CONECT    3   16   82
CONECT    4    6   79
CONECT    5   13   49
CONECT    6    4   11   32
CONECT    7    9   10  130
CONECT    8    2   23   28
CONECT    9    7   23   50
CONECT   10    7   28   51
CONECT   11    6   15   17
CONECT   12   19   32   52   53
CONECT   13    5   33   35
CONECT   14   16   26   37
CONECT   15   11   23   54   55
CONECT   16    3   14   19
CONECT   17   11   38   74
CONECT   18   21   30   57
CONECT   19   12   16   27
CONECT   20   24   36   40
CONECT   21   18   40  143
CONECT   22   33   45   67   68
CONECT   23    8    9   15
CONECT   24   20   28   58   59
CONECT   25   27   37  117
CONECT   26   14   35   69   70
CONECT   27   19   25   61
CONECT   28    8   10   24
CONECT   29   34   35   62
CONECT   30   18   36   41
CONECT   31   33   34   72
CONECT   32    6   12   39
CONECT   33   13   22   31
CONECT   34   29   31   91
CONECT   35   13   26   29
CONECT   36    1   20   30
CONECT   37   14   25   63
CONECT   38   17   39  104
CONECT   39   32   38   64
CONECT   40   20   21   73
CONECT   41   30   47   65   66
CONECT   42   43   47   75
CONECT   43   42   44  156
CONECT   44   43   45   71
CONECT   45   22   44   46
CONECT   46   45   47   48
CONECT   47   41   42   46
CONECT   48   46   81
CONECT   49    5   76   77  169
CONECT   50    9
```

```
CONECT   51   10
CONECT   52   12
CONECT   53   12
CONECT   54   15
CONECT   55   15
CONECT   56   79
CONECT   57   18
CONECT   58   24
CONECT   59   24
CONECT   60   82
CONECT   61   27
CONECT   62   29
CONECT   63   37
CONECT   64   39
CONECT   65   41
CONECT   66   41
CONECT   67   22
CONECT   68   22
CONECT   69   26
CONECT   70   26
CONECT   71   44
CONECT   72   31
CONECT   73   40
CONECT   74   17
CONECT   75   42
CONECT   76   49
CONECT   77   49
CONECT   78    2   84   90  249
CONECT   79    4   56   85  201
CONECT   80    1   83   86  233
CONECT   81   48   87   88  185
CONECT   82    3   60   89  217
CONECT   83   80
CONECT   84   78
CONECT   85   79
CONECT   86   80
CONECT   87   81
CONECT   88   81
CONECT   89   82
CONECT   90   78
CONECT   91   34   92   96  100
CONECT   92   91   93   94   95
CONECT   93   92
CONECT   94   92
CONECT   95   92
CONECT   96   91   97   98   99
CONECT   97   96
CONECT   98   96
CONECT   99   96
CONECT  100   91  101  102  103
CONECT  101  100
CONECT  102  100
CONECT  103  100
CONECT  104   38  105  109  113
CONECT  105  104  106  107  108
CONECT  106  105
CONECT  107  105
CONECT  108  105
CONECT  109  104  110  111  112
CONECT  110  109
CONECT  111  109
CONECT  112  109
```

```
CONECT  113  104  114  115  116
CONECT  114  113
CONECT  115  113
CONECT  116  113
CONECT  117   25  118  122  126
CONECT  118  117  119  120  121
CONECT  119  118
CONECT  120  118
CONECT  121  118
CONECT  122  117  123  124  125
CONECT  123  122
CONECT  124  122
CONECT  125  122
CONECT  126  117  127  128  129
CONECT  127  126
CONECT  128  126
CONECT  129  126
CONECT  130    7  131  135  139
CONECT  131  130  132  133  134
CONECT  132  131
CONECT  133  131
CONECT  134  131
CONECT  135  130  136  137  138
CONECT  136  135
CONECT  137  135
CONECT  138  135
CONECT  139  130  140  141  142
CONECT  140  139
CONECT  141  139
CONECT  142  139
CONECT  143   21  144  148  152
CONECT  144  143  145  146  147
CONECT  145  144
CONECT  146  144
CONECT  147  144
CONECT  148  143  149  150  151
CONECT  149  148
CONECT  150  148
CONECT  151  148
CONECT  152  143  153  154  155
CONECT  153  152
CONECT  154  152
CONECT  155  152
CONECT  156   43  157  161  165
CONECT  157  156  158  159  160
CONECT  158  157
CONECT  159  157
CONECT  160  157
CONECT  161  156  162  163  164
CONECT  162  161
CONECT  163  161
CONECT  164  161
CONECT  165  156  166  167  168
CONECT  166  165
CONECT  167  165
CONECT  168  165
CONECT  169   49  170  171  172
CONECT  170  169
CONECT  171  169
CONECT  172  169  173  174  175
CONECT  173  172
CONECT  174  172
```

```
CONECT  175  172  176  177  178
CONECT  176  175
CONECT  177  175
CONECT  178  175  179  180  181
CONECT  179  178
CONECT  180  178
CONECT  181  178  182  183  184
CONECT  182  181
CONECT  183  181
CONECT  184  181
CONECT  185   81  186  187  188
CONECT  186  185
CONECT  187  185
CONECT  188  185  189  190  191
CONECT  189  188
CONECT  190  188
CONECT  191  188  192  193  194
CONECT  192  191
CONECT  193  191
CONECT  194  191  195  196  197
CONECT  195  194
CONECT  196  194
CONECT  197  194  198  199  200
CONECT  198  197
CONECT  199  197
CONECT  200  197
CONECT  201   79  202  203  204
CONECT  202  201
CONECT  203  201
CONECT  204  201  205  206  207
CONECT  205  204
CONECT  206  204
CONECT  207  204  208  209  210
CONECT  208  207
CONECT  209  207
CONECT  210  207  211  212  213
CONECT  211  210
CONECT  212  210
CONECT  213  210  214  215  216
CONECT  214  213
CONECT  215  213
CONECT  216  213
CONECT  217   82  218  219  220
CONECT  218  217
CONECT  219  217
CONECT  220  217  221  222  223
CONECT  221  220
CONECT  222  220
CONECT  223  220  224  225  226
CONECT  224  223
CONECT  225  223
CONECT  226  223  227  228  229
CONECT  227  226
CONECT  228  226
CONECT  229  226  230  231  232
CONECT  230  229
CONECT  231  229
CONECT  232  229
CONECT  233   80  234  235  236
CONECT  234  233
CONECT  235  233
CONECT  236  233  237  238  239
```

```
CONECT  237  236
CONECT  238  236
CONECT  239  236  240  241  242
CONECT  240  239
CONECT  241  239
CONECT  242  239  243  244  245
CONECT  243  242
CONECT  244  242
CONECT  245  242  246  247  248
CONECT  246  245
CONECT  247  245
CONECT  248  245
CONECT  249   78  250  251  252
CONECT  250  249
CONECT  251  249
CONECT  252  249  253  254  255
CONECT  253  252
CONECT  254  252
CONECT  255  252  256  257  258
CONECT  256  255
CONECT  257  255
CONECT  258  255  259  260  261
CONECT  259  258
CONECT  260  258
CONECT  261  258  262  263  264
CONECT  262  261
CONECT  263  261
CONECT  264  261
CONECT  265  266  275  276  294
CONECT  266  265  270  273  274
CONECT  267  268  272  286
CONECT  268  267  269  277
CONECT  269  268  270  278
CONECT  270  266  269  271
CONECT  271  270  272  279
CONECT  272  267  271  280
CONECT  273  266
CONECT  274  266
CONECT  275  265
CONECT  276  265
CONECT  277  268
CONECT  278  269
CONECT  279  271
CONECT  280  272
CONECT  281  294
CONECT  282  294
CONECT  283  284  288  289
CONECT  284  283  285  290
CONECT  285  284  286  291
CONECT  286  267  285  287
CONECT  287  286  288  292
CONECT  288  283  287  293
CONECT  289  283
CONECT  290  284
CONECT  291  285
CONECT  292  287
CONECT  293  288
CONECT  294  265  281  282  298
CONECT  295  296  300  308
CONECT  296  295  297  301
CONECT  297  296  298  302
CONECT  298  294  297  299
```

```
CONECT  299  298  300  303
CONECT  300  295  299  304
CONECT  301  296
CONECT  302  297
CONECT  303  299
CONECT  304  300
CONECT  305  306  310  311
CONECT  306  305  307  312
CONECT  307  306  308  313
CONECT  308  295  307  309
CONECT  309  308  310  314
CONECT  310  305  309  315
CONECT  311  305
CONECT  312  306
CONECT  313  307
CONECT  314  309
CONECT  315  310
MASTER        0    0    0    0    0    0    0    3  315    0  315    0
END
```

Atomic coordinates of **2**$^{+}$$\subset$**1**$^{1,2,3\text{-}alt}$

```
HETATM    1  O1  UNK     1       2.652  -1.985  -3.479  1.00  0.00           O
HETATM    2  O2  UNK     1      -0.813  -4.930  -0.850  1.00  0.00           O
HETATM    3  O3  UNK     1      -2.769   0.633   2.449  1.00  0.00           O
HETATM    4  O4  UNK     1      -1.916  -2.720   2.278  1.00  0.00           O
HETATM    5  O5  UNK     1       0.583   2.781   0.627  1.00  0.00           O
HETATM    6  C6  UNK     1      -1.080  -3.042   3.343  1.00  0.00           C
HETATM    7  C7  UNK     1       3.196  -4.468   0.355  1.00  0.00           C
HETATM    8  C8  UNK     1       0.497  -4.706  -0.433  1.00  0.00           C
HETATM    9  C9  UNK     1       2.174  -4.635   1.295  1.00  0.00           C
HETATM   10  C10 UNK     1       2.812  -4.340  -0.987  1.00  0.00           C
HETATM   11  C11 UNK     1      -0.237  -4.166   3.230  1.00  0.00           C
HETATM   12  C12 UNK     1      -2.019  -1.050   4.650  1.00  0.00           C
HETATM   13  C13 UNK     1      -0.035   4.024   0.494  1.00  0.00           C
HETATM   14  C14 UNK     1      -1.447   2.501   3.243  1.00  0.00           C
HETATM   15  C15 UNK     1      -0.243  -5.049   1.988  1.00  0.00           C
HETATM   16  C16 UNK     1      -1.887   1.171   3.389  1.00  0.00           C
HETATM   17  C17 UNK     1       0.590  -4.476   4.312  1.00  0.00           C
HETATM   18  C18 UNK     1      -0.684  -0.857  -4.570  1.00  0.00           C
HETATM   19  C19 UNK     1      -1.465   0.364   4.461  1.00  0.00           C
HETATM   20  C20 UNK     1       0.523  -3.112  -3.436  1.00  0.00           C
HETATM   21  C21 UNK     1      -1.465  -1.997  -4.353  1.00  0.00           C
HETATM   22  C22 UNK     1       1.466   4.485  -1.528  1.00  0.00           C
HETATM   23  C23 UNK     1       0.827  -4.759   0.933  1.00  0.00           C
HETATM   24  C24 UNK     1       1.156  -4.390  -2.893  1.00  0.00           C
HETATM   25  C25 UNK     1      -0.206   2.287   5.344  1.00  0.00           C
HETATM   26  C26 UNK     1      -1.961   3.326   2.071  1.00  0.00           C
HETATM   27  C27 UNK     1      -0.619   0.947   5.410  1.00  0.00           C
HETATM   28  C28 UNK     1       1.483  -4.449  -1.404  1.00  0.00           C
HETATM   29  C29 UNK     1      -1.641   5.664   1.219  1.00  0.00           C
HETATM   30  C30 UNK     1       0.686  -0.810  -4.269  1.00  0.00           C
HETATM   31  C31 UNK     1      -0.146   6.182  -0.566  1.00  0.00           C
HETATM   32  C32 UNK     1      -1.068  -2.233   4.491  1.00  0.00           C
HETATM   33  C33 UNK     1       0.414   4.902  -0.509  1.00  0.00           C
```

```
HETATM   34  C34 UNK     1      -1.151   6.610   0.313  1.00  0.00           C
HETATM   35  C35 UNK     1      -1.127   4.361   1.316  1.00  0.00           C
HETATM   36  C36 UNK     1       1.287  -1.963  -3.729  1.00  0.00           C
HETATM   37  C37 UNK     1      -0.637   3.042   4.247  1.00  0.00           C
HETATM   38  C38 UNK     1       0.637  -3.700   5.482  1.00  0.00           C
HETATM   39  C39 UNK     1      -0.201  -2.584   5.538  1.00  0.00           C
HETATM   40  C40 UNK     1      -0.838  -3.098  -3.752  1.00  0.00           C
HETATM   41  C41 UNK     1       1.536   0.418  -4.613  1.00  0.00           C
HETATM   42  C42 UNK     1      -0.031   2.386  -4.993  1.00  0.00           C
HETATM   43  C43 UNK     1      -0.578   3.640  -4.675  1.00  0.00           C
HETATM   44  C44 UNK     1      -0.102   4.267  -3.520  1.00  0.00           C
HETATM   45  C45 UNK     1       0.925   3.723  -2.736  1.00  0.00           C
HETATM   46  C46 UNK     1       1.480   2.490  -3.123  1.00  0.00           C
HETATM   47  C47 UNK     1       0.969   1.778  -4.228  1.00  0.00           C
HETATM   48  O48 UNK     1       2.576   1.981  -2.436  1.00  0.00           O
HETATM   49  C49 UNK     1       1.560   2.699   1.718  1.00  0.00           C
HETATM   50  H50 UNK     1       2.422  -4.694   2.348  1.00  0.00           H
HETATM   51  H51 UNK     1       3.563  -4.179  -1.752  1.00  0.00           H
HETATM   52  H52 UNK     1      -2.437  -1.102   5.663  1.00  0.00           H
HETATM   53  H53 UNK     1      -2.844  -1.176   3.955  1.00  0.00           H
HETATM   54  H54 UNK     1      -1.214  -4.991   1.498  1.00  0.00           H
HETATM   55  H55 UNK     1      -0.121  -6.088   2.322  1.00  0.00           H
HETATM   56  H56 UNK     1      -3.825  -2.961   3.078  1.00  0.00           H
HETATM   57  H57 UNK     1      -1.137   0.023  -5.006  1.00  0.00           H
HETATM   58  H58 UNK     1       0.481  -5.213  -3.142  1.00  0.00           H
HETATM   59  H59 UNK     1       2.085  -4.581  -3.436  1.00  0.00           H
HETATM   60  H60 UNK     1      -4.350   1.663   1.590  1.00  0.00           H
HETATM   61  H61 UNK     1      -0.308   0.341   6.255  1.00  0.00           H
HETATM   62  H62 UNK     1      -2.471   5.921   1.867  1.00  0.00           H
HETATM   63  H63 UNK     1      -0.349   4.083   4.154  1.00  0.00           H
HETATM   64  H64 UNK     1      -0.222  -1.974   6.434  1.00  0.00           H
HETATM   65  H65 UNK     1       1.709   0.417  -5.698  1.00  0.00           H
HETATM   66  H66 UNK     1       2.505   0.291  -4.134  1.00  0.00           H
HETATM   67  H67 UNK     1       2.221   3.864  -1.053  1.00  0.00           H
HETATM   68  H68 UNK     1       1.970   5.392  -1.884  1.00  0.00           H
HETATM   69  H69 UNK     1      -2.316   2.626   1.311  1.00  0.00           H
HETATM   70  H70 UNK     1      -2.862   3.852   2.409  1.00  0.00           H
HETATM   71  H71 UNK     1      -0.512   5.223  -3.217  1.00  0.00           H
HETATM   72  H72 UNK     1       0.236   6.868  -1.318  1.00  0.00           H
HETATM   73  H73 UNK     1      -1.407  -3.998  -3.549  1.00  0.00           H
HETATM   74  H74 UNK     1       1.204  -5.369   4.242  1.00  0.00           H
HETATM   75  H75 UNK     1      -0.371   1.875  -5.889  1.00  0.00           H
HETATM   76  H76 UNK     1       1.600   1.638   1.952  1.00  0.00           H
HETATM   77  H77 UNK     1       1.148   3.219   2.583  1.00  0.00           H
HETATM   78  C78 UNK     1      -1.123  -6.330  -1.011  1.00  0.00           C
HETATM   79  C79 UNK     1      -3.190  -3.397   2.294  1.00  0.00           C
HETATM   80  C80 UNK     1       3.455  -2.316  -4.630  1.00  0.00           C
HETATM   81  C81 UNK     1       3.831   2.449  -2.977  1.00  0.00           C
HETATM   82  C82 UNK     1      -4.135   1.143   2.528  1.00  0.00           C
HETATM   83  H83 UNK     1       3.230  -1.624  -5.453  1.00  0.00           H
HETATM   84  H84 UNK     1      -0.390  -6.792  -1.687  1.00  0.00           H
HETATM   85  H85 UNK     1      -3.042  -4.454   2.546  1.00  0.00           H
HETATM   86  H86 UNK     1       3.201  -3.327  -4.978  1.00  0.00           H
HETATM   87  H87 UNK     1       3.877   2.209  -4.049  1.00  0.00           H
HETATM   88  H88 UNK     1       3.883   3.543  -2.885  1.00  0.00           H
HETATM   89  H89 UNK     1      -4.184   1.871   3.343  1.00  0.00           H
HETATM   90  H90 UNK     1      -1.036  -6.841  -0.042  1.00  0.00           H
HETATM   91 C109 UNK     1      -1.688   8.051   0.234  1.00  0.00           C
HETATM   92 C110 UNK     1      -2.772   8.331   1.291  1.00  0.00           C
HETATM   93 H111 UNK     1      -3.652   7.693   1.155  1.00  0.00           H
HETATM   94 H112 UNK     1      -2.397   8.184   2.309  1.00  0.00           H
HETATM   95 H113 UNK     1      -3.107   9.369   1.210  1.00  0.00           H
```

```
HETATM   96 C114 UNK      1       -0.522   9.043   0.467  1.00  0.00           C
HETATM   97 H115 UNK      1       -0.071   8.893   1.453  1.00  0.00           H
HETATM   98 H116 UNK      1        0.266   8.930  -0.282  1.00  0.00           H
HETATM   99 H117 UNK      1       -0.885  10.075   0.413  1.00  0.00           H
HETATM  100 C118 UNK      1       -2.300   8.303  -1.165  1.00  0.00           C
HETATM  101 H119 UNK      1       -2.672   9.331  -1.238  1.00  0.00           H
HETATM  102 H120 UNK      1       -1.567   8.159  -1.964  1.00  0.00           H
HETATM  103 H121 UNK      1       -3.141   7.627  -1.354  1.00  0.00           H
HETATM  104 C122 UNK      1        1.554  -4.119   6.647  1.00  0.00           C
HETATM  105 C123 UNK      1        1.503  -3.121   7.819  1.00  0.00           C
HETATM  106 H124 UNK      1        1.818  -2.117   7.514  1.00  0.00           H
HETATM  107 H125 UNK      1        0.500  -3.050   8.254  1.00  0.00           H
HETATM  108 H126 UNK      1        2.180  -3.451   8.613  1.00  0.00           H
HETATM  109 C127 UNK      1        1.109  -5.505   7.173  1.00  0.00           C
HETATM  110 H128 UNK      1        0.077  -5.472   7.539  1.00  0.00           H
HETATM  111 H129 UNK      1        1.163  -6.272   6.395  1.00  0.00           H
HETATM  112 H130 UNK      1        1.752  -5.823   8.000  1.00  0.00           H
HETATM  113 C131 UNK      1        3.018  -4.206   6.155  1.00  0.00           C
HETATM  114 H132 UNK      1        3.678  -4.502   6.977  1.00  0.00           H
HETATM  115 H133 UNK      1        3.138  -4.944   5.356  1.00  0.00           H
HETATM  116 H134 UNK      1        3.365  -3.241   5.773  1.00  0.00           H
HETATM  117 C135 UNK      1        0.663   2.878   6.472  1.00  0.00           C
HETATM  118 C136 UNK      1       -0.097   2.768   7.816  1.00  0.00           C
HETATM  119 H137 UNK      1       -1.041   3.321   7.779  1.00  0.00           H
HETATM  120 H138 UNK      1       -0.328   1.729   8.071  1.00  0.00           H
HETATM  121 H139 UNK      1        0.508   3.183   8.629  1.00  0.00           H
HETATM  122 C140 UNK      1        1.001   4.362   6.234  1.00  0.00           C
HETATM  123 H141 UNK      1        0.101   4.983   6.184  1.00  0.00           H
HETATM  124 H142 UNK      1        1.612   4.737   7.061  1.00  0.00           H
HETATM  125 H143 UNK      1        1.572   4.511   5.312  1.00  0.00           H
HETATM  126 C144 UNK      1        1.991   2.092   6.569  1.00  0.00           C
HETATM  127 H145 UNK      1        2.615   2.495   7.374  1.00  0.00           H
HETATM  128 H146 UNK      1        1.824   1.031   6.778  1.00  0.00           H
HETATM  129 H147 UNK      1        2.555   2.167   5.633  1.00  0.00           H
HETATM  130 C148 UNK      1        4.686  -4.537   0.745  1.00  0.00           C
HETATM  131 C149 UNK      1        5.186  -5.976   0.461  1.00  0.00           C
HETATM  132 H150 UNK      1        5.070  -6.234  -0.597  1.00  0.00           H
HETATM  133 H151 UNK      1        6.246  -6.073   0.720  1.00  0.00           H
HETATM  134 H152 UNK      1        4.624  -6.710   1.048  1.00  0.00           H
HETATM  135 C153 UNK      1        4.910  -4.228   2.239  1.00  0.00           C
HETATM  136 H154 UNK      1        4.453  -4.981   2.888  1.00  0.00           H
HETATM  137 H155 UNK      1        5.982  -4.226   2.460  1.00  0.00           H
HETATM  138 H156 UNK      1        4.507  -3.249   2.514  1.00  0.00           H
HETATM  139 C157 UNK      1        5.532  -3.547  -0.084  1.00  0.00           C
HETATM  140 H158 UNK      1        6.570  -3.563   0.264  1.00  0.00           H
HETATM  141 H159 UNK      1        5.547  -3.805  -1.146  1.00  0.00           H
HETATM  142 H160 UNK      1        5.150  -2.527   0.011  1.00  0.00           H
HETATM  143 C161 UNK      1       -2.930  -2.107  -4.821  1.00  0.00           C
HETATM  144 C162 UNK      1       -3.470  -0.780  -5.388  1.00  0.00           C
HETATM  145 H163 UNK      1       -2.910  -0.459  -6.272  1.00  0.00           H
HETATM  146 H164 UNK      1       -4.513  -0.908  -5.694  1.00  0.00           H
HETATM  147 H165 UNK      1       -3.438   0.026  -4.648  1.00  0.00           H
HETATM  148 C166 UNK      1       -3.009  -3.177  -5.938  1.00  0.00           C
HETATM  149 H167 UNK      1       -2.373  -2.906  -6.786  1.00  0.00           H
HETATM  150 H168 UNK      1       -2.687  -4.160  -5.580  1.00  0.00           H
HETATM  151 H169 UNK      1       -4.038  -3.273  -6.302  1.00  0.00           H
HETATM  152 C170 UNK      1       -3.843  -2.535  -3.650  1.00  0.00           C
HETATM  153 H171 UNK      1       -4.873  -2.666  -4.000  1.00  0.00           H
HETATM  154 H172 UNK      1       -3.523  -3.479  -3.201  1.00  0.00           H
HETATM  155 H173 UNK      1       -3.854  -1.773  -2.864  1.00  0.00           H
HETATM  156 C174 UNK      1       -1.636   4.273  -5.599  1.00  0.00           C
HETATM  157 C175 UNK      1       -1.034   4.455  -7.013  1.00  0.00           C
```

HETATM	158	H176	UNK	1	-0.157	5.110	-6.985	1.00	0.00	H
HETATM	159	H177	UNK	1	-0.725	3.502	-7.452	1.00	0.00	H
HETATM	160	H178	UNK	1	-1.772	4.905	-7.685	1.00	0.00	H
HETATM	161	C179	UNK	1	-2.875	3.351	-5.686	1.00	0.00	C
HETATM	162	H180	UNK	1	-2.623	2.367	-6.091	1.00	0.00	H
HETATM	163	H181	UNK	1	-3.325	3.204	-4.699	1.00	0.00	H
HETATM	164	H182	UNK	1	-3.633	3.794	-6.342	1.00	0.00	H
HETATM	165	C183	UNK	1	-2.099	5.655	-5.098	1.00	0.00	C
HETATM	166	H184	UNK	1	-2.841	6.066	-5.789	1.00	0.00	H
HETATM	167	H185	UNK	1	-2.568	5.598	-4.111	1.00	0.00	H
HETATM	168	H186	UNK	1	-1.270	6.367	-5.044	1.00	0.00	H
HETATM	169	C187	UNK	1	2.969	3.210	1.414	1.00	0.00	C
HETATM	170	H188	UNK	1	3.630	2.674	2.108	1.00	0.00	H
HETATM	171	H189	UNK	1	3.265	2.871	0.416	1.00	0.00	H
HETATM	172	C190	UNK	1	3.221	4.714	1.596	1.00	0.00	C
HETATM	173	H191	UNK	1	2.588	5.304	0.926	1.00	0.00	H
HETATM	174	H192	UNK	1	2.925	5.001	2.616	1.00	0.00	H
HETATM	175	C193	UNK	1	4.690	5.094	1.374	1.00	0.00	C
HETATM	176	H194	UNK	1	5.325	4.502	2.048	1.00	0.00	H
HETATM	177	H195	UNK	1	4.988	4.808	0.354	1.00	0.00	H
HETATM	178	C196	UNK	1	4.981	6.585	1.583	1.00	0.00	C
HETATM	179	H197	UNK	1	4.347	7.176	0.909	1.00	0.00	H
HETATM	180	H198	UNK	1	4.686	6.870	2.602	1.00	0.00	H
HETATM	181	C199	UNK	1	6.451	6.951	1.356	1.00	0.00	C
HETATM	182	H200	UNK	1	7.106	6.401	2.041	1.00	0.00	H
HETATM	183	H201	UNK	1	6.766	6.711	0.334	1.00	0.00	H
HETATM	184	H202	UNK	1	6.626	8.019	1.515	1.00	0.00	H
HETATM	185	C203	UNK	1	4.979	1.789	-2.231	1.00	0.00	C
HETATM	186	H204	UNK	1	4.896	2.031	-1.165	1.00	0.00	H
HETATM	187	H205	UNK	1	4.878	0.700	-2.320	1.00	0.00	H
HETATM	188	C206	UNK	1	6.352	2.234	-2.754	1.00	0.00	C
HETATM	189	H207	UNK	1	6.422	2.024	-3.831	1.00	0.00	H
HETATM	190	H208	UNK	1	6.447	3.324	-2.650	1.00	0.00	H
HETATM	191	C209	UNK	1	7.520	1.553	-2.030	1.00	0.00	C
HETATM	192	H210	UNK	1	7.439	1.743	-0.950	1.00	0.00	H
HETATM	193	H211	UNK	1	7.437	0.464	-2.154	1.00	0.00	H
HETATM	194	C212	UNK	1	8.897	2.014	-2.522	1.00	0.00	C
HETATM	195	H213	UNK	1	8.976	1.828	-3.602	1.00	0.00	H
HETATM	196	H214	UNK	1	8.981	3.102	-2.395	1.00	0.00	H
HETATM	197	C215	UNK	1	10.058	1.324	-1.799	1.00	0.00	C
HETATM	198	H216	UNK	1	10.025	1.520	-0.721	1.00	0.00	H
HETATM	199	H217	UNK	1	10.024	0.238	-1.940	1.00	0.00	H
HETATM	200	H218	UNK	1	11.025	1.676	-2.170	1.00	0.00	H
HETATM	201	C219	UNK	1	-3.838	-3.255	0.927	1.00	0.00	C
HETATM	202	H220	UNK	1	-3.923	-2.185	0.696	1.00	0.00	H
HETATM	203	H221	UNK	1	-3.171	-3.690	0.173	1.00	0.00	H
HETATM	204	C222	UNK	1	-5.224	-3.913	0.865	1.00	0.00	C
HETATM	205	H223	UNK	1	-5.127	-4.988	1.071	1.00	0.00	H
HETATM	206	H224	UNK	1	-5.859	-3.509	1.665	1.00	0.00	H
HETATM	207	C225	UNK	1	-5.928	-3.719	-0.483	1.00	0.00	C
HETATM	208	H226	UNK	1	-6.037	-2.643	-0.683	1.00	0.00	H
HETATM	209	H227	UNK	1	-5.292	-4.112	-1.287	1.00	0.00	H
HETATM	210	C228	UNK	1	-7.308	-4.385	-0.558	1.00	0.00	C
HETATM	211	H229	UNK	1	-7.199	-5.462	-0.368	1.00	0.00	H
HETATM	212	H230	UNK	1	-7.941	-3.998	0.252	1.00	0.00	H
HETATM	213	C231	UNK	1	-8.008	-4.172	-1.903	1.00	0.00	C
HETATM	214	H232	UNK	1	-8.165	-3.106	-2.104	1.00	0.00	H
HETATM	215	H233	UNK	1	-7.414	-4.580	-2.728	1.00	0.00	H
HETATM	216	H234	UNK	1	-8.987	-4.660	-1.926	1.00	0.00	H
HETATM	217	C235	UNK	1	-5.147	0.028	2.753	1.00	0.00	C
HETATM	218	H236	UNK	1	-5.045	-0.716	1.955	1.00	0.00	H
HETATM	219	H237	UNK	1	-4.930	-0.484	3.697	1.00	0.00	H

```
HETATM  220 C238 UNK     1      -6.586   0.564   2.779  1.00  0.00           C
HETATM  221 H239 UNK     1      -6.684   1.308   3.583  1.00  0.00           H
HETATM  222 H240 UNK     1      -6.795   1.101   1.843  1.00  0.00           H
HETATM  223 C241 UNK     1      -7.635  -0.537   2.979  1.00  0.00           C
HETATM  224 H242 UNK     1      -7.543  -1.277   2.171  1.00  0.00           H
HETATM  225 H243 UNK     1      -7.421  -1.078   3.912  1.00  0.00           H
HETATM  226 C244 UNK     1      -9.075  -0.011   3.020  1.00  0.00           C
HETATM  227 H245 UNK     1      -9.169   0.723   3.831  1.00  0.00           H
HETATM  228 H246 UNK     1      -9.287   0.536   2.091  1.00  0.00           H
HETATM  229 C247 UNK     1     -10.115  -1.119   3.210  1.00  0.00           C
HETATM  230 H248 UNK     1     -10.071  -1.850   2.394  1.00  0.00           H
HETATM  231 H249 UNK     1      -9.951  -1.660   4.148  1.00  0.00           H
HETATM  232 H250 UNK     1     -11.131  -0.712   3.236  1.00  0.00           H
HETATM  233 C251 UNK     1       4.923  -2.229  -4.245  1.00  0.00           C
HETATM  234 H252 UNK     1       5.135  -1.213  -3.886  1.00  0.00           H
HETATM  235 H253 UNK     1       5.109  -2.901  -3.398  1.00  0.00           H
HETATM  236 C254 UNK     1       5.860  -2.579  -5.408  1.00  0.00           C
HETATM  237 H255 UNK     1       5.636  -3.593  -5.766  1.00  0.00           H
HETATM  238 H256 UNK     1       5.660  -1.907  -6.255  1.00  0.00           H
HETATM  239 C257 UNK     1       7.345  -2.492  -5.034  1.00  0.00           C
HETATM  240 H258 UNK     1       7.569  -1.478  -4.673  1.00  0.00           H
HETATM  241 H259 UNK     1       7.547  -3.166  -4.190  1.00  0.00           H
HETATM  242 C260 UNK     1       8.289  -2.836  -6.193  1.00  0.00           C
HETATM  243 H261 UNK     1       8.063  -3.848  -6.554  1.00  0.00           H
HETATM  244 H262 UNK     1       8.086  -2.161  -7.036  1.00  0.00           H
HETATM  245 C263 UNK     1       9.769  -2.747  -5.812  1.00  0.00           C
HETATM  246 H264 UNK     1      10.033  -1.737  -5.478  1.00  0.00           H
HETATM  247 H265 UNK     1      10.011  -3.438  -4.996  1.00  0.00           H
HETATM  248 H266 UNK     1      10.415  -2.995  -6.660  1.00  0.00           H
HETATM  249 C267 UNK     1      -2.531  -6.473  -1.566  1.00  0.00           C
HETATM  250 H268 UNK     1      -3.234  -5.972  -0.889  1.00  0.00           H
HETATM  251 H269 UNK     1      -2.591  -5.947  -2.528  1.00  0.00           H
HETATM  252 C270 UNK     1      -2.942  -7.940  -1.751  1.00  0.00           C
HETATM  253 H271 UNK     1      -2.219  -8.443  -2.408  1.00  0.00           H
HETATM  254 H272 UNK     1      -2.887  -8.459  -0.784  1.00  0.00           H
HETATM  255 C273 UNK     1      -4.351  -8.104  -2.334  1.00  0.00           C
HETATM  256 H274 UNK     1      -5.076  -7.596  -1.682  1.00  0.00           H
HETATM  257 H275 UNK     1      -4.404  -7.589  -3.305  1.00  0.00           H
HETATM  258 C276 UNK     1      -4.772  -9.567  -2.515  1.00  0.00           C
HETATM  259 H277 UNK     1      -4.044 -10.075  -3.162  1.00  0.00           H
HETATM  260 H278 UNK     1      -4.723 -10.079  -1.544  1.00  0.00           H
HETATM  261 C279 UNK     1      -6.176  -9.723  -3.105  1.00  0.00           C
HETATM  262 H280 UNK     1      -6.931  -9.254  -2.464  1.00  0.00           H
HETATM  263 H281 UNK     1      -6.245  -9.255  -4.093  1.00  0.00           H
HETATM  264 H282 UNK     1      -6.446 -10.778  -3.219  1.00  0.00           H
HETATM  265  N91 UNK     1      -0.801   0.132   0.116  1.00  0.00           N
HETATM  266  C92 UNK     1       0.168  -1.060   0.176  1.00  0.00           C
HETATM  267  C93 UNK     1       3.918  -0.309   2.233  1.00  0.00           C
HETATM  268  C94 UNK     1       2.770  -0.703   2.943  1.00  0.00           C
HETATM  269  C95 UNK     1       1.563  -0.935   2.289  1.00  0.00           C
HETATM  270  C96 UNK     1       1.457  -0.781   0.901  1.00  0.00           C
HETATM  271  C97 UNK     1       2.598  -0.403   0.180  1.00  0.00           C
HETATM  272  C98 UNK     1       3.803  -0.169   0.838  1.00  0.00           C
HETATM  273  H99 UNK     1      -0.402  -1.871   0.633  1.00  0.00           H
HETATM  274 H100 UNK     1       0.374  -1.321  -0.863  1.00  0.00           H
HETATM  275 H101 UNK     1      -0.284   1.031   0.174  1.00  0.00           H
HETATM  276 H102 UNK     1      -1.444   0.118   0.926  1.00  0.00           H
HETATM  277 H103 UNK     1       2.824  -0.856   4.016  1.00  0.00           H
HETATM  278 H104 UNK     1       0.704  -1.249   2.868  1.00  0.00           H
HETATM  279 H105 UNK     1       2.550  -0.280  -0.898  1.00  0.00           H
HETATM  280 H106 UNK     1       4.663   0.151   0.261  1.00  0.00           H
HETATM  281 H107 UNK     1      -1.980  -0.955  -1.237  1.00  0.00           H
```

```
HETATM  282 H108 UNK     1      -0.917   0.243  -1.976  1.00  0.00           H
HETATM  283 C283 UNK     1       7.637   0.494   4.249  1.00  0.00           C
HETATM  284 C284 UNK     1       7.638  -0.053   2.965  1.00  0.00           C
HETATM  285 C285 UNK     1       6.435  -0.316   2.312  1.00  0.00           C
HETATM  286 C286 UNK     1       5.202  -0.040   2.929  1.00  0.00           C
HETATM  287 C287 UNK     1       5.219   0.509   4.223  1.00  0.00           C
HETATM  288 C288 UNK     1       6.422   0.772   4.875  1.00  0.00           C
HETATM  289 H289 UNK     1       8.575   0.699   4.757  1.00  0.00           H
HETATM  290 H290 UNK     1       8.578  -0.283   2.472  1.00  0.00           H
HETATM  291 H291 UNK     1       6.454  -0.766   1.324  1.00  0.00           H
HETATM  292 H292 UNK     1       4.282   0.750   4.716  1.00  0.00           H
HETATM  293 H293 UNK     1       6.410   1.203   5.872  1.00  0.00           H
HETATM  294 C294 UNK     1      -1.625   0.075  -1.163  1.00  0.00           C
HETATM  295 C295 UNK     1      -4.948   2.860  -1.392  1.00  0.00           C
HETATM  296 C296 UNK     1      -3.621   3.296  -1.557  1.00  0.00           C
HETATM  297 C297 UNK     1      -2.554   2.405  -1.483  1.00  0.00           C
HETATM  298 C298 UNK     1      -2.772   1.048  -1.214  1.00  0.00           C
HETATM  299 C299 UNK     1      -4.092   0.604  -1.056  1.00  0.00           C
HETATM  300 C300 UNK     1      -5.162   1.492  -1.150  1.00  0.00           C
HETATM  301 H301 UNK     1      -3.423   4.340  -1.775  1.00  0.00           H
HETATM  302 H302 UNK     1      -1.547   2.771  -1.657  1.00  0.00           H
HETATM  303 H303 UNK     1      -4.287  -0.447  -0.861  1.00  0.00           H
HETATM  304 H304 UNK     1      -6.174   1.124  -1.013  1.00  0.00           H
HETATM  305 C305 UNK     1      -8.232   5.625  -1.626  1.00  0.00           C
HETATM  306 C306 UNK     1      -7.032   6.008  -1.024  1.00  0.00           C
HETATM  307 C307 UNK     1      -5.967   5.111  -0.950  1.00  0.00           C
HETATM  308 C308 UNK     1      -6.082   3.813  -1.476  1.00  0.00           C
HETATM  309 C309 UNK     1      -7.296   3.442  -2.078  1.00  0.00           C
HETATM  310 C310 UNK     1      -8.359   4.339  -2.154  1.00  0.00           C
HETATM  311 H311 UNK     1      -9.061   6.324  -1.684  1.00  0.00           H
HETATM  312 H312 UNK     1      -6.927   7.004  -0.604  1.00  0.00           H
HETATM  313 H313 UNK     1      -5.047   5.408  -0.456  1.00  0.00           H
HETATM  314 H314 UNK     1      -7.397   2.453  -2.517  1.00  0.00           H
HETATM  315 H315 UNK     1      -9.286   4.037  -2.632  1.00  0.00           H
CONECT    1   36   80
CONECT    2    8   78
CONECT    3   16   82
CONECT    4    6   79
CONECT    5   13   49
CONECT    6    4   11   32
CONECT    7    9   10  130
CONECT    8    2   23   28
CONECT    9    7   23   50
CONECT   10    7   28   51
CONECT   11    6   15   17
CONECT   12   19   32   52   53
CONECT   13    5   33   35
CONECT   14   16   26   37
CONECT   15   11   23   54   55
CONECT   16    3   14   19
CONECT   17   11   38   74
CONECT   18   21   30   57
CONECT   19   12   16   27
CONECT   20   24   36   40
CONECT   21   18   40  143
CONECT   22   33   45   67   68
CONECT   23    8    9   15
CONECT   24   20   28   58   59
CONECT   25   27   37  117
CONECT   26   14   35   69   70
CONECT   27   19   25   61
CONECT   28    8   10   24
```

```
CONECT   29   34   35   62
CONECT   30   18   36   41
CONECT   31   33   34   72
CONECT   32    6   12   39
CONECT   33   13   22   31
CONECT   34   29   31   91
CONECT   35   13   26   29
CONECT   36    1   20   30
CONECT   37   14   25   63
CONECT   38   17   39  104
CONECT   39   32   38   64
CONECT   40   20   21   73
CONECT   41   30   47   65   66
CONECT   42   43   47   75
CONECT   43   42   44  156
CONECT   44   43   45   71
CONECT   45   22   44   46
CONECT   46   45   47   48
CONECT   47   41   42   46
CONECT   48   46   81
CONECT   49    5   76   77  169
CONECT   50    9
CONECT   51   10
CONECT   52   12
CONECT   53   12
CONECT   54   15
CONECT   55   15
CONECT   56   79
CONECT   57   18
CONECT   58   24
CONECT   59   24
CONECT   60   82
CONECT   61   27
CONECT   62   29
CONECT   63   37
CONECT   64   39
CONECT   65   41
CONECT   66   41
CONECT   67   22
CONECT   68   22
CONECT   69   26
CONECT   70   26
CONECT   71   44
CONECT   72   31
CONECT   73   40
CONECT   74   17
CONECT   75   42
CONECT   76   49
CONECT   77   49
CONECT   78    2   84   90  249
CONECT   79    4   56   85  201
CONECT   80    1   83   86  233
CONECT   81   48   87   88  185
CONECT   82    3   60   89  217
CONECT   83   80
CONECT   84   78
CONECT   85   79
CONECT   86   80
CONECT   87   81
CONECT   88   81
CONECT   89   82
CONECT   90   78
```

```
CONECT   91   34   92   96  100
CONECT   92   91   93   94   95
CONECT   93   92
CONECT   94   92
CONECT   95   92
CONECT   96   91   97   98   99
CONECT   97   96
CONECT   98   96
CONECT   99   96
CONECT  100   91  101  102  103
CONECT  101  100
CONECT  102  100
CONECT  103  100
CONECT  104   38  105  109  113
CONECT  105  104  106  107  108
CONECT  106  105
CONECT  107  105
CONECT  108  105
CONECT  109  104  110  111  112
CONECT  110  109
CONECT  111  109
CONECT  112  109
CONECT  113  104  114  115  116
CONECT  114  113
CONECT  115  113
CONECT  116  113
CONECT  117   25  118  122  126
CONECT  118  117  119  120  121
CONECT  119  118
CONECT  120  118
CONECT  121  118
CONECT  122  117  123  124  125
CONECT  123  122
CONECT  124  122
CONECT  125  122
CONECT  126  117  127  128  129
CONECT  127  126
CONECT  128  126
CONECT  129  126
CONECT  130    7  131  135  139
CONECT  131  130  132  133  134
CONECT  132  131
CONECT  133  131
CONECT  134  131
CONECT  135  130  136  137  138
CONECT  136  135
CONECT  137  135
CONECT  138  135
CONECT  139  130  140  141  142
CONECT  140  139
CONECT  141  139
CONECT  142  139
CONECT  143   21  144  148  152
CONECT  144  143  145  146  147
CONECT  145  144
CONECT  146  144
CONECT  147  144
CONECT  148  143  149  150  151
CONECT  149  148
CONECT  150  148
CONECT  151  148
CONECT  152  143  153  154  155
```

```
CONECT  153  152
CONECT  154  152
CONECT  155  152
CONECT  156   43  157  161  165
CONECT  157  156  158  159  160
CONECT  158  157
CONECT  159  157
CONECT  160  157
CONECT  161  156  162  163  164
CONECT  162  161
CONECT  163  161
CONECT  164  161
CONECT  165  156  166  167  168
CONECT  166  165
CONECT  167  165
CONECT  168  165
CONECT  169   49  170  171  172
CONECT  170  169
CONECT  171  169
CONECT  172  169  173  174  175
CONECT  173  172
CONECT  174  172
CONECT  175  172  176  177  178
CONECT  176  175
CONECT  177  175
CONECT  178  175  179  180  181
CONECT  179  178
CONECT  180  178
CONECT  181  178  182  183  184
CONECT  182  181
CONECT  183  181
CONECT  184  181
CONECT  185   81  186  187  188
CONECT  186  185
CONECT  187  185
CONECT  188  185  189  190  191
CONECT  189  188
CONECT  190  188
CONECT  191  188  192  193  194
CONECT  192  191
CONECT  193  191
CONECT  194  191  195  196  197
CONECT  195  194
CONECT  196  194
CONECT  197  194  198  199  200
CONECT  198  197
CONECT  199  197
CONECT  200  197
CONECT  201   79  202  203  204
CONECT  202  201
CONECT  203  201
CONECT  204  201  205  206  207
CONECT  205  204
CONECT  206  204
CONECT  207  204  208  209  210
CONECT  208  207
CONECT  209  207
CONECT  210  207  211  212  213
CONECT  211  210
CONECT  212  210
CONECT  213  210  214  215  216
CONECT  214  213
```

```
CONECT  215  213
CONECT  216  213
CONECT  217   82  218  219  220
CONECT  218  217
CONECT  219  217
CONECT  220  217  221  222  223
CONECT  221  220
CONECT  222  220
CONECT  223  220  224  225  226
CONECT  224  223
CONECT  225  223
CONECT  226  223  227  228  229
CONECT  227  226
CONECT  228  226
CONECT  229  226  230  231  232
CONECT  230  229
CONECT  231  229
CONECT  232  229
CONECT  233   80  234  235  236
CONECT  234  233
CONECT  235  233
CONECT  236  233  237  238  239
CONECT  237  236
CONECT  238  236
CONECT  239  236  240  241  242
CONECT  240  239
CONECT  241  239
CONECT  242  239  243  244  245
CONECT  243  242
CONECT  244  242
CONECT  245  242  246  247  248
CONECT  246  245
CONECT  247  245
CONECT  248  245
CONECT  249   78  250  251  252
CONECT  250  249
CONECT  251  249
CONECT  252  249  253  254  255
CONECT  253  252
CONECT  254  252
CONECT  255  252  256  257  258
CONECT  256  255
CONECT  257  255
CONECT  258  255  259  260  261
CONECT  259  258
CONECT  260  258
CONECT  261  258  262  263  264
CONECT  262  261
CONECT  263  261
CONECT  264  261
CONECT  265  266  275  276  294
CONECT  266  265  270  273  274
CONECT  267  268  272  286
CONECT  268  267  269  277
CONECT  269  268  270  278
CONECT  270  266  269  271
CONECT  271  270  272  279
CONECT  272  267  271  280
CONECT  273  266
CONECT  274  266
CONECT  275  265
CONECT  276  265
```

```
CONECT  277  268
CONECT  278  269
CONECT  279  271
CONECT  280  272
CONECT  281  294
CONECT  282  294
CONECT  283  284  288  289
CONECT  284  283  285  290
CONECT  285  284  286  291
CONECT  286  267  285  287
CONECT  287  286  288  292
CONECT  288  283  287  293
CONECT  289  283
CONECT  290  284
CONECT  291  285
CONECT  292  287
CONECT  293  288
CONECT  294  265  281  282  298
CONECT  295  296  300  308
CONECT  296  295  297  301
CONECT  297  296  298  302
CONECT  298  294  297  299
CONECT  299  298  300  303
CONECT  300  295  299  304
CONECT  301  296
CONECT  302  297
CONECT  303  299
CONECT  304  300
CONECT  305  306  310  311
CONECT  306  305  307  312
CONECT  307  306  308  313
CONECT  308  295  307  309
CONECT  309  308  310  314
CONECT  310  305  309  315
CONECT  311  305
CONECT  312  306
CONECT  313  307
CONECT  314  309
CONECT  315  310
MASTER         0    0    0    0    0    0    0    3  315    0  315    0
END
```
